# Entropy‐Engineered Multi‐Metallic MOFs Unlock High‐Performance Ammonium‐Ion Storage

**DOI:** 10.1002/smll.74541

**Published:** 2026-07-27

**Authors:** Barathi Palani, Ľubica Cenknerová, Martin Pumera

**Affiliations:** ^1^ Advanced Nanorobots and Multiscale Robotics Laboratory Faculty of Electrical Engineering and Computer Science VSB – Technical University of Ostrava Ostrava Czech Republic; ^2^ Analytical Laboratory Institute of Organic Chemistry and Biochemistry of the Czech Academy of Sciences Praha Czech Republic; ^3^ Future Energy and Innovation Laboratory Central European Institute of Technology Brno University of Technology Brno Czech Republic; ^4^ Department of Medical Research China Medical University Hospital China Medical University No. 91 Hsueh‐Shih Road Taichung Taiwan; ^5^ Office of Research and Development Asia University Taichung Taiwan; ^6^ Department of Chemical and Biomolecular Engineering Yonsei University Seodaemun‐gu Seoul South Korea

**Keywords:** ammonium‐ion storage, aqueous ammonium‐ion batteries, entropy engineering, hydrogen‐bonding interactions, metal–organic frameworks

## Abstract

Aqueous ammonium ion batteries (AAIBs) are an emerging sustainable energy technology owing to their high ionic conductivity, safety, low cost, and non‐metallic charge carrier. However, their practical applicability is restricted by low energy density, a narrower potential window, and poor cycling stability. An entropy‐engineered amino group‐functionalized metal–organic framework (EnMOF‐NH_2_) is reported as a promising cathode for high‐performance AAIBs. EnMOF‐NH_2_ consists of five metal nodes (Co, Ni, Fe, Mn, Mo) and an amino‐functionalized benzene dicarboxylic acid linker (BDC‐NH_2_), which provides multiple redox centers, enhanced structural stability, and abundant hydrogen bonding sites for efficient NH_4_^+^ storage. The incorporation of Mo metal nodes into the multi‐metallic MOF can strengthen the metal‐ligand interaction and suppress the metal dissolution, while amino‐functionalized BDC provides polarity to the framework and enables fast NH_4_^+^ storage through hydrogen bonding. EnMOF‐NH_2_ achieves a high reversible capacity of 130.8 mAh g^−^^1^ at 0.3 A g^−^^1^ in 1.0 M (NH_4_)_2_SO_4_, outperforming the non‐amino‐functionalized EnMOF‐BDC. It exhibits excellent rate capability, and capacity activation to 162% after 2500 cycles. A full cell coupled with an EnMOF‐NH_2_ cathode and perylene tetracarboxylic dianhydride (PTCDA) anode retains 83% capacity over 1000 cycles. Such entropy‐engineered, amino‐group‐functionalized MOF materials represent a promising cathode for future aqueous ammonium‐ion batteries.

## Graphical abstract

Entropy‐engineered amino‐functionalized EnMOF‐NH_2_ cathode with five metal nodes (Co, Ni, Fe, Mn, Mo) enables NH_4_^+^ hydrogen‐bonding storage and multi‐metal redox activity, while suppressing metal dissolution.

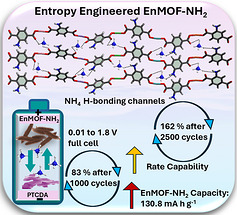

## 1 Introduction

Aqueous rechargeable batteries have shown significant interest for sustainable energy storage devices owing to their low cost, low flammability, safety, and environmental friendliness [1, 2, 3, 4, 5, 6, 7, 8]. Aqueous ammonium ion batteries (AAIBs) are showing immense attention to researchers compared to conventional metal‐ion batteries, because of their high ionic conductivity, small hydrated ionic radii (0.331 nm), low molecular mass (18 g mol^−1^), non‐metallic NH_4_^+^ ion storage characteristics, and environmentally benign ammonium salts [9, 10, 11]. Moreover, the tetrahedral geometry of the NH_4_^+^ ion forms hydrated species that enable hydrogen‐bonding interactions with host materials, resulting in rapid NH_4_^+^ transport and pseudocapacitive charge storage. However, the practical application of AAIBs is limited by their low energy density and narrow operating potential range in aqueous electrolyte. Therefore, the research should focus on host materials to deliver high capacity, a wide cell‐voltage window, and robust structural stability upon repeated NH_4_^+^ insertion/extraction.

Various promising cathode materials, including Prussian blue analogues, transition‐metal oxides, MXenes, and organic molecules, have been investigated for AAIBs [12, 13]. However, they suffer from sluggish solid‐state diffusion, insufficient redox utilization, or rapid capacity fading in aqueous electrolytes. For instance, Wu et al. reported (NH_4_)_1.47_Ni[Fe(CN)_6_]_0.88_ as a cathode material for NH_4_^+^ storage, which displayed a capacity of 60 mA h g^−1^ at 0.15 A g^−1^, and its capacity decays to 74% after 2000 cycles at 0.3 A g^−1^. Han et al. reported polyaniline intercalated V_2_O_5_ as a promising cathode with a high discharge capacity of 307 mA h g^−1^ at 0.5 A g^−1^; however, their capacity decayed to 42% in 1000 cycles at 5 A g^−1^. These challenges are likely due to structural degradation and metal dissolution during continuous cycling, which can lead to electrolyte discoloration and increased polarization. On the other hand, various organic and inorganic hosts, such as covalent organic frameworks, Perylene‐3,4,9,10‐tetracarboxylic dianhydride (PTCDA), quinones, transition‐metal oxides, and, chalcogenides, have been reported as high‐performance anode materials owing to their well‐defined redox peaks in NH_4_^+^ electrolytes, enabling NH_4_^+^/H^+^ storage at their active sites [14]. The reversible and stable redox characteristics of anode materials enable their integration with cathodes to construct high‐performance full cells with a wide cell‐voltage range.

Metal–organic frameworks (MOFs) are outstanding multifunctional materials composed of metal nodes bridged by organic ligands [15, 16, 17, 18, 19, 20, 21, 22]. Owing to their inherent tunability, high porosity, large surface area, and high conductivity, MOFs are widely studied in many fields, including ammonia gas storage [23, 24], electrocatalysis [25, 26], and electrochemical energy storage [16, 27, 28], However, it demonstrated versatility; its structural and chemical properties should be engineered to enhance its performance in energy storage. Interestingly, a high‐entropy strategy provides a new pathway by incorporating multiple metal cations into a single coordination lattice, thereby facilitating abundant active sites, a stable porous framework, and improved conductivity through a high‐entropy “cocktail” effect [29, 30, 31]. The entropy‐driven MOFs exhibit high catalytic activity owing to their high porosity, which enables efficient adsorption and desorption [32, 33, 34]. Their stable frameworks provide wide channels that facilitate the insertion and extraction of large‐sized Na^+^, and K^+^ batteries and zinc air batteries, thereby improving specific capacity and cycling stability [35, 36, 37]. In addition to cation entropy in MOF materials, an amino‐functionalized linker can enhance MOF performance in catalysis and energy storage applications [38, 39, 40]. For instance, 2‐amino, 1,4 benzene dicarboxylic acid linker (BDC‐NH_2_) based MOF, such as NiNH_2_‐BDC and UiO‐66‐NH_2_, displays high specific capacity, catalytic activity, and cycling stability compared to a non‐amino functionalized BDC‐based MOF [41, 42]. This NH_2_ functional group increases the polarity of the MOF framework and hydrogen‐bonding sites, thereby facilitating rapid charge‐transfer processes during electrocatalysis and charge storage.

Inspired by entropy engineering, incorporating multiple metal centers into the MOF is another effective strategy to enhance its stability, porosity, and catalytic activity, owing to their multi‐redox sites and tunable coordination environments [43]. In this work, we designed an entropy‐inspired multi‐metal MOF (EnMOF‐NH_2_) with Fe, Ni, Co, Mn, and Mo as metal nodes and an amino‐functionalized BDC linker to improve their electrochemical performance. The introduction of Mo metal (from the 4d transition) into the 3d transition metal nodes can strengthen the metal‐ligand bonding and stabilize the framework due to their stable Mo redox centers (Mo^6^^+^/Mo^5^^+^), which broaden the accessible redox active sites and facilitate the rate capability and cycling stability in energy storage applications [32, 44]. On the other hand, the amino‐functionalized BDC‐NH_2_ ligand provides more coordinated networks, increases the polarity, and introduces additional N─H···N and N─H···O hydrogen bonding sites [41, 42]. These characteristics strengthen the interaction of MOFs with polar ammonium ions in the electrolyte during discharge, thereby improving charge storage compared to that of non‐amino‐functionalized BDC‐based MOFs. Therefore, this multi‐redox activity of the metals and the amino‐functionalized ligand improves the structure‐property relationship of multifunctional MOFs, which enhances the battery performance.

Guided by these considerations, we have demonstrated EnMOF‐NH_2_ as a promising cathode derived from high‐entropy metal nodes (CoNiFeMnMo) and BDC‐NH_2_ linker, for high‐performance AAIBs. The EnMOF‐NH_2_ displayed discharge capacity of 130.8 mAh g^−1^ at a current density of 0.3 A g^−1^ in 1.0 M (NH_4_)_2_SO_4_ aqueous electrolyte, whereas EnMOF‐BDC derived from the same metal nodes and BDC linker (without amino functionalization) displayed discharge capacity of 80.1 mAh g^−1^ at the same current density. Interestingly, the EnMOF‐NH_2_ exhibits excellent rate capability and enhanced cycling performance, increasing to 162% after 2500 cycles. It is worth noting that Mo‐free MOF (CoNiFeMn‐NH_2_) shows a low discharge capacity of 91.6 mAh g^−1^, and capacity decay and significant metal dissolution into the electrolyte, which emphasizes the role of Mo‐anchored entropy engineering in stabilizing the EnMOF‐NH_2_ framework. Furthermore, we conducted various electrochemical experiments and physicochemical characterizations to elucidate the ammonium ion storage mechanism in EnMOF‐NH_2_. To investigate its practical applicability in AAIBs, we assembled a full cell by pairing EnMOF‐NH_2_ with a PTCDA anode in a rocking‐chair configuration. The full cell provides an extended operating potential in the window of 0.01–1.8 V and displays a specific capacity of 59.4 mAh g^−1^ at a current density of 0.4 A g^−1^ and retains 83% of its initial capacity after 1000 cycles with nearly 100% coulombic efficiency. This entropy‐inspired multi‐metallic MOF with an amino‐functionalized BDC linker is a promising cathode material for advancing next‐generation AAIBs in real‐time applications.

## 2 Results and Discussion

### 2.1 Synthesis and Characterization of MOF

**FIGURE 1:**
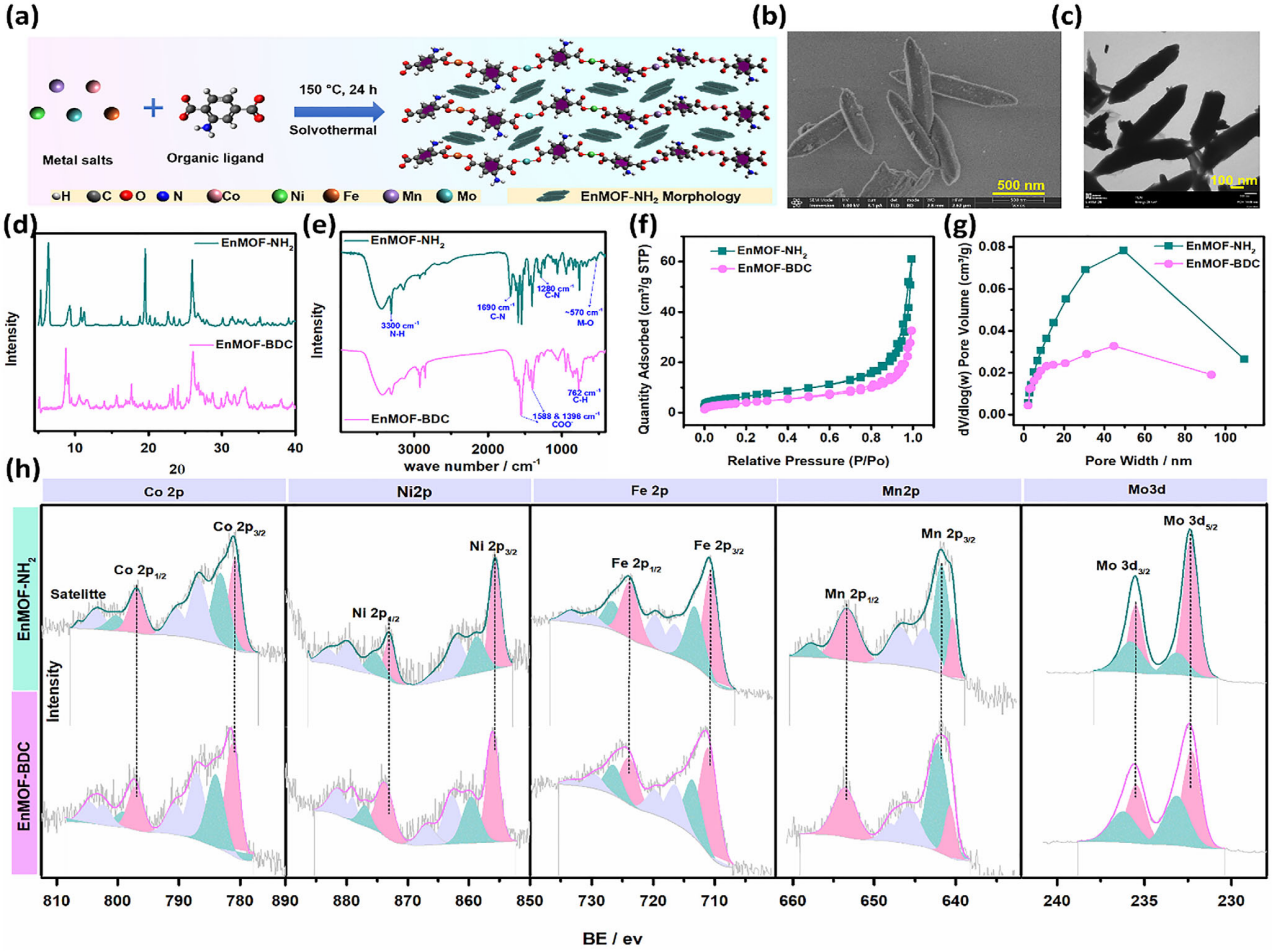
Schematic synthetic procedure, morphology and structure analysis. (a) Schematic illustration of EnMOF‐NH_2_ synthesis; (b) SEM; and (c) TEM image of EnMOF‐NH_2_; (d) XRD pattern; (e) FT‐IR; (f) BET nitrogen physisorption isotherms; (g) BJH desorption pore‐size distributions; and (h) XPS spectra of EnMOF‐NH_2_ and EnMOF‐BDC.

A one‐step solvothermal approach has been employed to synthesize EnMOF‐NH_2_ and EnMOF‐BDC, as shown in Figure 1a. Equimolar ratios of Co, Ni, Fe, Mn, and Mo are chosen as metal nodes, and BDC and BDC‐NH_2_ are chosen as linkers for the entropy engineering of MOF. The equimolar incorporation of five different metal species maximizes the configurational entropy in the MOF framework. The calculated configurational entropy (ΔS_conf_) is 1.609 R, enabling entropy engineering of the MOF framework to tune its structural and electrochemical properties. The detailed preparation procedure is given in the experimental section. Figure 1b,c and Figure S1 display scanning electron microscopy (SEM), and transmission electron microscopy (TEM) images of EnMOF‐NH_2_ and EnMOF‐BDC. SEM and TEM images clearly demonstrate that linker functionalization can distinguish the morphology. The non‐functionalized EnMOF‐BDC shows an intergrown plate‐like structure with dense agglomeration, which can be ascribed to planar stacking and π‐π interactions of the BDC linker (Figure S1). In contrast, the EnMOF‐NH_2_ shows a rod‐like morphology, where the NH_2_ functional group introduces additional hydrogen bonding and steric hindrance that might disrupt the planar stacking and promote a rod‐like structure. Such a linker‐dependent morphology has been studied in previous reports [45]. Scanning transmission electron microscopy‐energy dispersive X‐ray spectroscopy (STEM‐EDS) analysis reveals the homogeneous distribution of Co, Ni, Fe, Mn, and Mo across both EnMOF‐NH_2_ and EnMOF‐BDC, evidencing successful incorporation of multiple metals into the framework (Figure S2 and Table S1). Notably, Mo exhibits a relatively higher EDS signal (26.7 at.% in EnMOF‐NH_2_; 29.1 at.% in EnMOF‐BDC), which is attributed to the higher charge density of Mo^6+^ and the stronger Mo─O bond than the 3d transition metal‐oxygen bonds (Co─O, Ni─O, Fe─O, Mn─O). This stronger metal‐ligand interaction enables Mo to act as a structural anchor, stabilizing adjacent 3d metal coordination centers and minimizing the lattice distortion arising from the multi‐metal driven configurational entropy [32].

The X‐ray diffraction (XRD) patterns of EnMOF‐NH_2_ and EnMOF‐BDC are represented in Figure 1d. Both samples show characteristic diffraction peaks at lower 2θ angles (∼5–12°), confirming the long‐range ordered BDC‐based MOF frameworks. EnMOF‐NH_2_ shows a prominent peak at 5.36°, 6.4°, 9.0°, 9.3°, 10.8°, 11.2°, 16.3°, 19.5°, 22.7°, 23.5°, 24.2°, 25.8° which are in agreement with reported aminated BDC‐based MOFs such as Ni‐NH_2_‐BDC, and MIL‐88B(Fe)‐NH_2_ [41, 46, 47, 48, 49, 50, 51, 52, 53], while EnMOF‐BDC shows peaks at 8.8°, 9.2°, 10.6°, 11.6°, 15.6°, 17.6°, 22.8°, 23.3°, 24.1°, 25.9°, which are closely related to BDC‐based MOFs [41, 46, 47, 49, 52, 53]. Interestingly, the EnMOF‐NH_2_ exhibits sharper and more intense diffraction peaks compared to the non‐functionalized EnMOF‐BDC, indicating enhanced crystallinity, which agrees with previous reports that amino functionalization strengthens the metal–ligand coordination and assists in forming long‐range ordered structures [54, 55]. It is worth noting that no additional distinct peaks related to crystalline oxide impurities, such as Fe_2_O_3_, Co_3_O_4_, MnO, and MoO_3_, were observed, confirming that metals are predominantly incorporated into the framework, rather than forming separate crystalline phases [56]. However, weak additional reflections were noticed in EnMOF‐BDC, indicating its lower crystallinity, similar to reported terephthalate‐based frameworks [57]. The diffraction results confirm the successful synthesis of multi‐metallic MOFs with both ligands, with high crystallinity in EnMOF‐NH_2_.

Fourier transform infrared (FTIR) spectra provide further confirmation for the coordination of metal centers with organic ligands. Both EnMOF‐NH_2_ and EnMOF‐BDC display characteristic vibrational bands in the 1000–2000 cm^−^^1^ range, indicating that the aromatic and carboxylate structure of the BDC linker is retained in the structure (Figure 1e). For EnMOF‐NH_2_, a vibrational band observed at 3309 cm^−^^1^, which is attributed to N─H stretching, reveals the incorporation of ‐NH_2_ groups in the framework. The absence of this feature in EnMOF‐BDC evidences the ligand‐dependent functionalization. The bands at 1588 and 1396 cm^−^^1^ are related to asymmetric and symmetric stretching modes of coordinated carboxylate (COO^−^), while the bands at 1690 and 1280 cm^−^^1^ are associated with C═O and C─N vibrations respectively, suggesting the coordination between the ligand and multi‐metal centers [41]. Furthermore, the band at ∼570 cm^−^^1^, assigned to the M─O stretching, confirms the existence of strong metal–ligand interactions. Overall, these spectral features confirm that amino functionalization is involved in the framework formation, with preserved structural integrity of MOF.

Brunauer–Emmett–Teller method (BET) measurements were carried out at 77 K to understand the textural properties of EnMOF‐NH_2_ and EnMOF‐BDC. Both samples exhibit type II hysteresis, indicating mesoporous‐macroporous structure‐based MOFs. EnMOF‐NH_2_ adsorbs more N_2_ (Figure 1f; Figure S3a,b), showing a more open framework and N_2_ accessibility than EnMOF‐BDC. Quantitative BET analysis of EnMOF‐NH_2_ and EnMOF‐BDC shows surface areas of 23.1 and 14.0 m^2^ g^−^^1^ and their corresponding external areas of 23.3 and 15.8 m^2^ g^−^^1^, respectively. The increased surface area of EnMOF‐NH_2_ may be attributed to amino functionalization, which preserves accessible pores without collapsing the structure [58].

The Barrett–Joyner–Halenda (BJH) desorption pore‐size distributions (Figure 1g; Table S2) show that EnMOF‐NH_2_ has maximum mesopores in the range of 3–10 nm, with a high cumulative pore volume (0.086 cm^3^ g^−^^1^) than that of EnMOF‐BDC (0.046 cm^3^ g^−^^1^). The t‐plot curves for both samples are linear (Figure S3c,d), confirming dominant mesoporosity with negligible microporosity [59]. The enhanced mesoporous framework and increased accessible surface area of EnMOF‐NH_2_ facilitate rapid ammonium‐ion transport and electrolyte infiltration, which are crucial for improving battery performance.

The X‐ray photoelectron spectroscopy (XPS) spectra of EnMOF‐NH_2_ and EnMOF‐BDC provide surface elemental compositions and the chemical states of metal centers and the influence of amino functionalization on MOF framework stability. The survey spectra of both EnMOF‐NH_2_ and EnMOF‐BDC exhibit distinct peaks related to the C 1s, O 1s, Co 2p, Ni 2p, Fe 2p, Mn 2p, and Mo 3d orbitals (Figure S4). XPS surface composition reveals higher Mo incorporation in both frameworks (EnMOF‐NH_2_: 29.57 at.%; EnMOF‐BDC: 22.94 at.%), consistent with STEM‐EDS. The presence of Mo^6+^ (Mo 3d_5/2_ at 232.4 eV) indicates strong metal‐ligand coordination, enabling it to act as a structural anchor within the multi‐metallic framework (Table S3). Notably, their distinct spin‐orbit doublet characteristics show the dominant surface oxidation states within the framework (Figure 1h). In the Co 2p spectrum, a peak at 781.2 eV (Co 2p_3/2_) and 797.0 eV (Co 2p_1/2_) with a spin‐orbit splitting (ΔE) of 15.8 eV associated with Co^2^^+^ species, which is in good agreement with reported Co‐based MOFs [60]. Similarly, the Ni 2p spectrum displayed peaks at 855.7 eV (Ni 2p_3/2_) and 873.8 eV (Ni 2p_1/2_), with ΔE of 18.1 eV, related to Ni^2^^+^ in octahedral coordination. The Fe 2p spectrum shows peaks at 710.7 eV (Fe 2p_3/2_) and 724.4 eV (Fe 2p_1/2_), with a ΔE of 13.7 eV, characteristic of Fe^2^^+^/Fe^3^^+^ species. The Mn 2p doublet observed at 641.3 eV and 653.3 eV with ΔE of 12.0 eV, related to Mn^2^^+^ oxidation states, whereas Mo 3d showed peaks at 232.4 eV (Mo 3d_5/2_) and 235.5 eV (Mo 3d_3/2_), confirming the existence of Mo^6^^+^ species with a 3.1 eV spin–orbit splitting. These binding energy separations are in good agreement with those of high‐entropy MOFs [61, 62]. In contrast, minor satellite and shoulder peaks are observed at higher binding energies for Co, Ni, and Fe, indicating surface‐limited oxidation, possibly due to air exposure during synthesis. However, XRD revealed no separate crystalline oxide phases in the bulk framework. Compared to EnMOF‐BDC, EnMOF‐NH_2_ showed a negative shift in binding energies (∼0.3–0.4 eV shift) at the 2p and 3d metal centers, indicating increased electron density at the metal centers due to the electron‐donating –NH_2_ substituent. Notably, EnMOF‐BDC shows slightly higher binding energies and enhanced surface oxidation, suggesting weaker coordination and partial surface reactivity.

Collectively, the structural characterization provides evidence for both the entropy‐engineered multi‐metallic framework and a structural anchor role of Mo. XRD confirms the formation of long‐range ordered BDC‐based MOF without detectable metal oxide impurities (*e.g*., Fe_2_O_3_, Co_3_O_4_, MnO, MoO_3_), indicating that all five metal species are incorporated into the coordination lattice. The calculated configurational entropy (ΔS_conf_ = 1.609 R) satisfies the thermodynamic criteria for entropy stabilization. STEM‐EDS (Table S1) mapping shows a uniform distribution of all five metals without phase segregation. XPS further confirms the presence of Mo^6^^+^ and its surface enrichment, indicating strong Mo‐O coordination. FTIR spectroscopy supports this with a distinct M‐O vibration (∼570 cm^−^^1^), confirming the metal‐ligand coordination. Most importantly, the ICP‐OES results (Table S5) provide direct evidence of Mo‐driven structural anchoring. The detailed discussion is given in Section 2.3. The Mo‐containing EnMOF‐NH_2_ shows significantly suppressed metal dissolution compared to the Mo‐free system (CoNiFeMn‐NH_2_), confirming that Mo incorporation reinforces the framework during repeated NH_4_^+^ insertion/extraction. Together, these results demonstrate that the entropy‐engineered multi‐metal incorporation and Mo‐induced structural stabilization synergistically govern the framework stability.

### 2.2 Electrochemical Performance of EnMOF‐NH_2_ and EnMOF‐BDC

**FIGURE 2:**
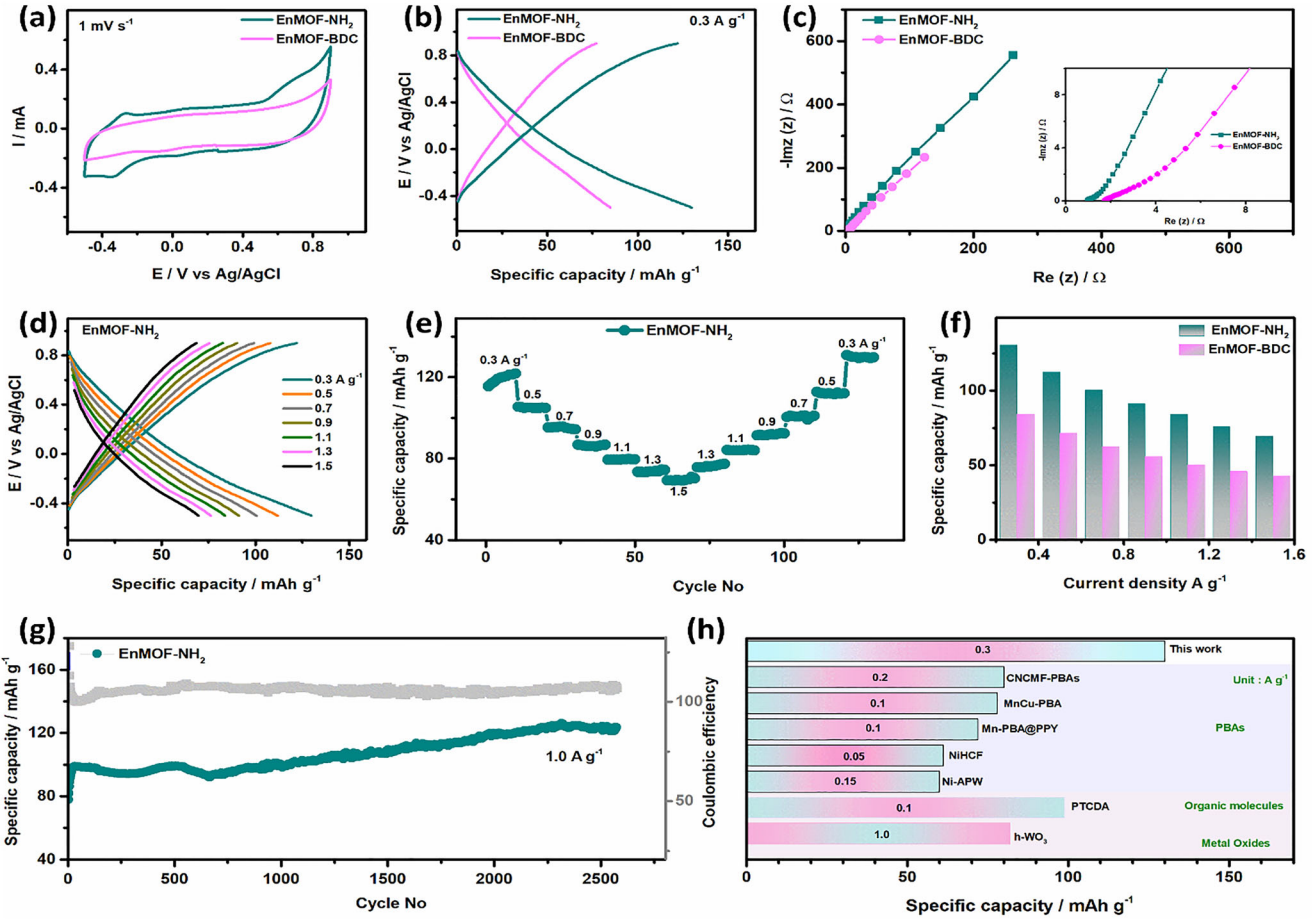
Comparative electrochemical performance of EnMOF‐NH_2_ and EnMOF‐BDC. Cyclic voltammograms (a), charge–discharge profiles (b), Nyquist plots (c) of EnMOF‐NH_2_ and EnMOF‐BDC; rate capability (d), capacity retention (e) at different current densities of EnMOF‐NH_2_; (f) capacity retention comparison of EnMOF‐NH_2_ and EnMOF‐BDC at different current densities; (g) cycling stability of EnMOF‐NH_2_; (h) specific capacity *vs* literature reports.

The electrochemical properties of the EnMOF‐NH_2_, and EnMOF‐BDC were studied using a three‐electrode configuration in the potential range of −0.9 to +0.5 V vs Ag/AgCl in 1 M (NH_4_)_2_SO_4_ aqueous electrolyte, with platinum and Ag/AgCl as counter and reference electrode, respectively. As shown in Figure 2a, EnMOF‐NH_2_ shows multiple distinct redox peaks at −0.34/−0.26 V; 0.03/0.05 and 0.32/0.65 V vs Ag/AgCl, corresponding to NH_4_^+^ insertion/extraction processes, related to multiple‐metal redox centers. It is difficult to distinguish the individual redox contributions of EnMOF‐NH_2_ because its multiphase MOF framework features transition metal centers with similar local chemical environments and oxidation states [63, 64]. The EnMOF‐NH_2_ displays redox peaks with higher current responses than EnMOF‐BDC, indicating high redox reversibility and improved interfacial charge transfer. This improved redox features and ion accessibility might be due to the electron‐donating nature of the amino group, which introduces hydrogen bonding sites and increases the local electron density at the metal‐oxygen nodes. A similar amino‐functionalization has been reported in MOF/reduced graphene oxide composite electrodes for high‐performance lithium‐ion batteries [65]. The galvanostatic charge–discharge (GCD) profiles (Figure 2b) further verify these observations. EnMOF‐NH_2_ delivers a discharge capacity of 130.8 mAh g^−^^1^ at a current density of 0.3 A g^−^^1^, which is higher than that of EnMOF‐BDC (∼80 mAh g^−^^1^). The higher reversible capacity can be attributed to the hydrogen‐bonding network between NH_4_^+^ and the amino linker of BDC, forming [EnMOF‐NH_2_^…^NH_4_] adducts that facilitate a proton‐coupled electron transfer (PCET) process [66, 67]. These interactions may stabilize the intermediate states that can lower the diffusion barriers and increase the charge‐transfer efficiency, resulting in improved redox reversibility and capacity. Figure 2c displays electrochemical impedance spectroscopy of EnMOF‐NH_2_ and EnMOF‐BDC electrodes. Both electrodes exhibit nearly linear profiles without a pronounced semicircle, indicating that ionic diffusion characteristics dominate over charge‐transfer resistance. The steeper Warburg region slope observed for EnMOF‐NH_2_ reveals facile NH_4_^+^ diffusion and improved surface adsorption within the porous framework. These results demonstrate that amino functionalization can enhance electronic delocalization and ion transport across the multi‐metal coordination network, consistent with improved redox kinetics observed in CV and GCD.

Figure 2d–g displays the rate capability and cycling performance of EnMOF‐NH_2_ and EnMOF‐BDC. As expected, capacity decreases as current density increases from 0.3 to 1.5 A g^−^^1^ (Figure 2d; Figure S5a). Interestingly, while returning to a low current density of 0.3 A g^−^^1^, the capacity increases beyond the initial value, indicating improved electrochemical activation and electrolyte infiltration with cycling (Figure 2e; Figure S5b). In contrast, the EnMOF‐BDC shows much lower specific capacity at the identical current densities (Figure 2f). Interestingly, the initial capacity is increased to 162% after 2500 cycles at a current density of 1.0 A g^−^^1^ (Figure 2g), while EnMOF‐BDC maintains 100% of its initial capacity with minimal improvement after 250 cycles at a current density of 0.3 A g^−^^1^ (Figure S5c). The specific capacity, rate capability, and cyclic performance of EnMOF‐NH_2_ are considerably better than those of the previously reported materials on NH_4_^+^ storage (Figure 2h, Table S4) [68, 69, 70, 71, 72, 73, 74].

This progressive activation of capacity is attributed to three synergistic processes in EnMOF‐NH_2_. First, the measured mesopore range of 3–10 nm in EnMOF‐NH_2_ may be partially blocked by residual DMF or synthesis solvent trapped within the framework during solvothermal preparation. During prolonged electrochemical cycling, this residual solvent may desorb, progressively opening inaccessible pore channels and exposing additional redox‐active sites to the electrolyte. This is consistent with a significantly high pore volume of EnMOF‐NH_2_ (0.086 cm^3^ g^−^^1^) compared to EnMOF‐BDC (0.046 cm^3^ g^−^^1^), which provides a larger active pore space. Such electrochemical activation behavior has been reported in the literature. Notably, the CNCMF‐PBA system listed in Table S4 exhibits an even more pronounced capacity of 223.5% upon cycling, confirming the progressive pore activation in the high entropy porous system. Second, the repeated NH_4_^+^ insertion/extraction process can induce mild structural relaxation at the metal nodes, thereby reducing diffusion barriers and enabling more efficient utilization of all five metal redox centers (Co, Ni, Fe, Mn, Mo). The ex situ XRD data show that the crystalline framework remains unchanged during charge‐discharge cycles, indicating that the capacity originates from activation. Third, the –NH_2_ groups on the BDC linker progressively form a strong hydrogen‐bonded adduct ([EnMOF‐NH_2_···NH_4_^+^]) upon continuous cycling, thereby increasing the density of NH_4_^+^ storage sites and lowering the desolvation energy barrier. In addition, Mo nodes play a crucial role by suppressing metal dissolution and supporting capacity retention over 2500 cycles (ICP‐OES, Table S5). The absence of such activation in EnMOF‐BDC might be due to a lack of –NH_2_ groups and lower mesoporous volume.

### 2.3 Role of Mo and Entropy‐Driven Stability

To clearly decouple the individual contributions of ‐NH_2_ functionalization, Mo incorporation, and configurational entropy, three independent control systems were systematically designed. (i) EnMOF‐BDC vs EnMOF‐NH_2_ to isolate the amino functionalization effect (identical metal nodes, linkers varied, discussed in previous section); (ii) CoNiFeMn‐NH_2_ vs. EnMOF‐NH_2_ to isolate the Mo contribution (identical BDC‐NH_2_ linker, Mo nodes removed); and (iii) binary, ternary, quaternary and quinary to quantify the entropy contribution (increasing metal complexity). The detailed results are discussed below.

**FIGURE 3:**
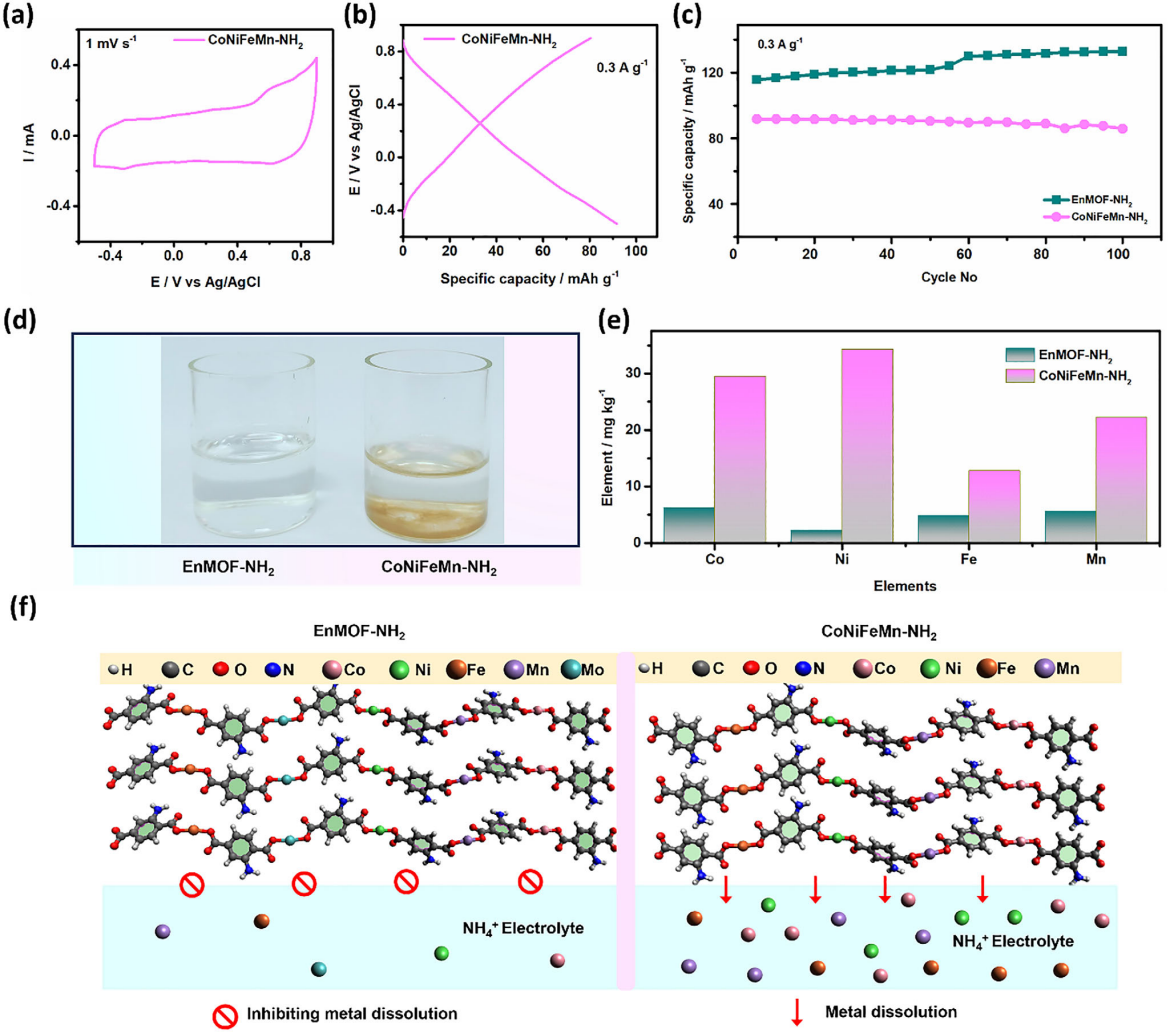
Role of Mo and Entropy‐Driven Stability. (a) CV curves and (b) GCD profiles of Mo‐free MOF (CoNiFeMn‐NH_2_) at a current density of 0.3 A g^−1^ in 1 M (NH_4_)_2_SO_4_ aqueous electrolyte; (c) capacity retention comparing CoNiFeMn‐NH_2_ to EnMOF‐NH_2._ (d) Digital photographs of (NH_4_)_2_SO_4_ electrolytes after 100 GCD cycles for EnMOF‐NH_2_ (colorless) and CoNiFeMn‐NH_2_ (discolored). (e) ICP‐OES metal dissolution after 100 cycles for EnMOF‐NH_2_ vs. CoNiFeMn‐NH_2_. (f) Schematic representation of suppressed metal dissolution and entropy‐driven stability in EnMOF‐NH_2_ vs. CoNiFeMn‐NH_2_.

To understand the role of Mo in improving the electrochemical characteristics, Mo‐containing EnMOF‐NH_2_ and Mo‐free CoNiFeMn‐NH_2_ were investigated. The EnMOF‐NH_2_ electrode exhibits a high reversible capacity of 130.8 mAh g^−^^1^ at current density of 0.3 A g^−^^1^ and long‐term stability compared with CoNiFeMn‐NH_2_ (91.6 mAh g^−^^1^, Figure 3a,b), which shows capacity decay with cycles (Figure 3c). In addition, the CoNiFeMn‐NH_2_ system showed visible discoloration of the electrolyte within a few charge‐discharge cycles (Figure 3d), whereas EnMOF‐NH_2_ cell retained a colorless electrolyte even after 2500 cycles. To further understand the role of Mo in stabilizing MOF framework, Inductively Coupled Plasma Optical Emission Spectrometry (ICP‐OES) was performed on the electrolytes from EnMOF‐NH_2_ and CoNiFeMn‐NH_2_ cells after 100 cycles (Table S5). ICP‐OES confirms that EnMOF‐NH_2_ shows minimal metal dissolution, whereas CoNiFeMn‐NH_2_ leaches high concentrations of metals into the electrolyte (Figure 3e). The strengthened Mo─O coordination in EnMOF‐NH_2_ stabilizes the configurational entropy during NH_4_^+^ insertion/extraction, thereby suppressing metal dissolution, as shown in Figure 3f. As a result, the adjacent 3d metal centers exhibit high redox utilization, resulting in improved capacity and cycling stability even after 2500 cycles.

To understand the entropy contribution, binary (CoMo‐, NiMo‐, FeMo‐NH_2_), ternary (CoFeMo‐, CoNiMo‐, NiFeMo‐NH_2_), and quaternary (CoNiFeMo‐NH_2_) systems were subjected to electrochemical measurements. Binary NH_2_‐MOFs, such as CoMo‐, NiMo‐, and FeMo‐NH_2_, exhibit specific capacities of 50.9, 51.1, and 71.2 mAh g^−1^ at 0.7 A g^−1^ (Figure S6), while ternary NH_2_‐MOFs, such as CoFeMo‐, CoNiMo‐, and NiFeMo‐NH_2_, display specific capacities of 64.2, 63.1, and 91.2 mAh g^−1^ at 0.7 A g^−1^ (Figure S7). The quaternary NH_2_‐MOFs, CoNiFeMo‐NH_2_ show the capacity of 94.8 mAh g^−1^ at 0.7 A g^−1^ (Figure S8). All systems exhibit good rate capability and recover their initial capacities upon returning to low current densities. The capacity increases monotonically at a consistent current density of 0.7 A g^−1^ from 50.9–71.2 mAh g^−^^1^ (binary), 63.1–91.2 mAh g^−^^1^ (ternary), 94.8 mAh g^−^^1^ (quaternary), and 101.2 mAh g^−^^1^ (quinary EnMOF‐NH_2,_ Figure 2d). These observations directly correlate with an increase in configurational entropy (ΔS_conf_) from 0.693 R (binary) to 1.609 R (quinary), confirming an entropy‐driven enhancement in electrochemical performance through the progressive addition of multi‐metal redox centers, thereby improving capacity and rate capability.

Interestingly, no visible colour changes were observed in the post‐cycled electrolytes of these binary, ternary, and quaternary NH_2_‐MOFs (data not shown), which confirms that Mo incorporation effectively minimizes metal dissolution and stabilizes the framework. The ICP and electrochemical studies reveal that Mo acts as a coordination anchor to stabilize the lattice without significant degradation of MOF framework during repetitive NH_4_^+^ insertion/extraction. Hence, the synergistic combination of these three contributions, such as amino group hydrogen bonding, Mo‐driven structural anchoring, and entropy‐stabilised multi‐metal redox, collectively provides high capacity, rate capability, and cycling stability of EnMOF‐NH_2_ compared to any single contributed control system. This entropy‐dependent enhancement of capacity and stability is consistent with recent studies on medium‐entropy Prussian blue analogs for ultralong‐life AAIBs, in which increased configurational entropy enhances redox utilization [61, 72].

### 2.4 Electrolyte and pH‐Dependent Behavior

To investigate the role of different cations on ammonium storage, the electrochemical performance of EnMOF‐NH_2_ was carried out in 1 M Li_2_SO_4_, Na_2_SO_4_, K_2_SO_4_, and (NH_4_)_2_SO_4_ aqueous electrolyte under identical experimental conditions. The typical CV of Li^+^, Na^+^, and K^+^‐ based electrolytes shows broad and feeble redox responses, whereas (NH_4_)_2_SO_4_ shows comparatively better CV response, which indicates improved interfacial kinetics and charge transfer (Figures S9 and S10). Interestingly, EnMOF‐NH_2_ in (NH_4_)_2_SO_4_ electrolyte displays a high specific capacity of 130.8 mAh g^−^^1^, which is significantly higher than that in the Li_2_SO_4_ (≈97.7 mAh g^−^^1^), Na_2_SO_4_ (≈92.5 mAh g^−^^1^), and K_2_SO_4_ (≈102.8 mAh g^−^^1^) under identical conditions. To support this observation, 1 M (NH_4_)_2_SO_4_ electrolyte shows better rate capability and higher recovered initial capacity when returning to low current density of 0.3 A g^−^^1^, indicating progressive utilization of redox sites during charge–discharge cycling. In contrast, the Li^+^‐, Na^+^‐, and K^+^‐based electrolytes failed to recover their original capacity when returning to low current density of 0.3 A g^−^^1^. The cycling stability of EnMOF‐NH_2_ electrode in (NH_4_)_2_SO_4_ shows 113.8% capacity retention after 100 cycles, while Li^+^‐, Na^+^‐, and K^+^‐based systems retain 81.7%, 96.4%, and 76.2% of their initial capacities, respectively.

The superior performance of NH_4_^+^ originates from three mechanistic advantages. First, NH_4_^+^ (0.331 nm) has a smaller hydrated ionic radius than Na^+^ (0.358 nm) and Li^+^ (0.382 nm), enabling faster NH_4_^+^ insertion kinetics and a lower desolvation barrier at the electrode‐electrolyte interface. Notably, the K^+^ (0.331 nm) has a similar hydrated ionic radius to NH_4_^+^ but delivers low capacity (102.8 vs. 130.8 mAh g^−^^1^), indicating that ionic size does not alone impact the performance. Second, the tetrahedral NH_4_^+^ ion has four N‐H bonds that simultaneously form multiple hydrogen bonds with –NH_2_ groups and carboxylate oxygen sites of the BDC‐NH_2_ linker. In contrast, a lack of hydrogen‐bonding capability with alkali metal ions leads to higher desolvation and slower charge‐transfer kinetics, resulting in lower capacity. Third, this hydrogen‐bonding network enables a proton‐coupled electron transfer (PCET) mechanism at the metal redox centers, thereby providing an additional charge‐storage pathway. These results suggest that the amino‐functionalized, entropy‐inspired EnMOF‐NH_2_ exhibits selective NH_4_^+^ storage capability compared to other alkali metal ions.

To understand the effect of anions on ammonium ion storage, the electrochemical performance of EnMOF‐NH_2_ was evaluated in NH_4_Cl, NH_4_OAc, and (NH_4_)_2_SO_4_ electrolytes under identical experimental conditions (Figure S11). Cyclic voltammograms of all three electrolytes exhibit similar pseudocapacitive features at a scan rate of 1 mV s^−1^. Among these, (NH_4_)_2_SO_4_ electrolyte exhibits high integrated area and current density, indicating improved charge transfer kinetics. The GCD studies reveal that EnMOF‐NH_2_ delivers a higher discharge capacity of 130.8 mAh g^−^^1^ at a current density of 0.3 A g^−^^1^ in (NH_4_)_2_SO_4_ electrolyte, compared with capacities of 112 and 107 mAh g^−^^1^ in NH_4_OAc and NH_4_Cl electrolytes, respectively, at the same current density of 0.3 A g^−^^1^. This capacity trend (NH_4_)_2_SO_4_> NH_4_OAc > NH_4_Cl might be due to strong electrostatic interactions and a hydrogen‐bonding network (NH_4_^+^···SO_4_^2^^−^···H_2_O), which may stabilize the EnMOF‐electrolyte interface, resulting in high capacity and stable cycling in (NH_4_)_2_SO_4_ electrolyte.

To understand the influence of pH on ammonium ion storage, the electrochemical performance of EnMOF‐NH_2_ was examined in (NH_4_)_2_SO_4_ electrolyte with pH values from 5 to 8 (Figure S12). The EnMOF‐NH_2_ shows a maximum capacity of 114.4 mAh g^−1^ at pH 5.8, which is the measured pH of the 1 M (NH_4_)_2_SO_4_ aqueous electrolyte. The excessive protonation at lower pH (pH 5) and partial deprotonation at higher pH (pH 7 and 8) disrupts the hydrogen bonding network between NH_4_^+^ and the –NH_2_/carboxylate sites of the BDC‐NH_2_ linker and weakens the metal‐ligand interaction, resulting in relatively lower ammonium ion storage. This observation suggests that near‐neutral pH conditions (pH ≈ 5.8) provide optimal amino‐group protonation and a stable hydrogen‐bond network, which enables highly reversible ammonium‐ion storage within the EnMOF‐NH_2_ framework.

To elucidate the charge‐storage mechanism, the EnMOF‐NH_2_ cathode was subjected to cyclic voltammetry at scan rates from 1 to 7 mV s^−1^ (Figure S13a). The peak current increases with preserved CV profile as the scan rate increases, indicating reversible charge transport and efficient electrode‐electrolyte interaction. The b value was calculated from the double logarithmic plot of peak current (log *i*) verses scan rate (log *v*) (Figure S13b), derived from the power law relationship *i*  =  *av^b^*. The redox peak current was recorded at the peak potential of ‐0.27 V vs Ag/AgCl. If the b value is close to 0.5, it corresponds to a diffusion‐controlled intercalation process, and a value close to 1.0 indicates a surface‐controlled capacitive process. The calculated b value for EnMOF‐NH_2_ is 0.7, indicating a mixed surface‐ and diffusion‐controlled charge‐storage mechanism. To further analyze the capacitive and diffusion‐controlled contributions, the scan rate effect is examined using Dunn's equation *i = k_1_v* + *k_2_v^1/2^*. Here, the kinetic components *k_1_v* and *k_2_v^1/2^* are related to capacitive and diffusion‐controlled contributions, respectively. The calculated capacitive contribution is 55% over the scan range of 1–7 mV s^−^^1^ (Figure S13c), confirming mixed surface‐ and diffusion‐controlled charge‐storage behavior within the MOF framework. Such mixed capacitive–diffusion kinetics have been reported in high‐entropy frameworks for Zn^2+^/NH_4_^+^ energy storage in aqueous media [61, 75].

### 2.5 Ammonium‐Ion Storage Mechanism

**FIGURE 4:**
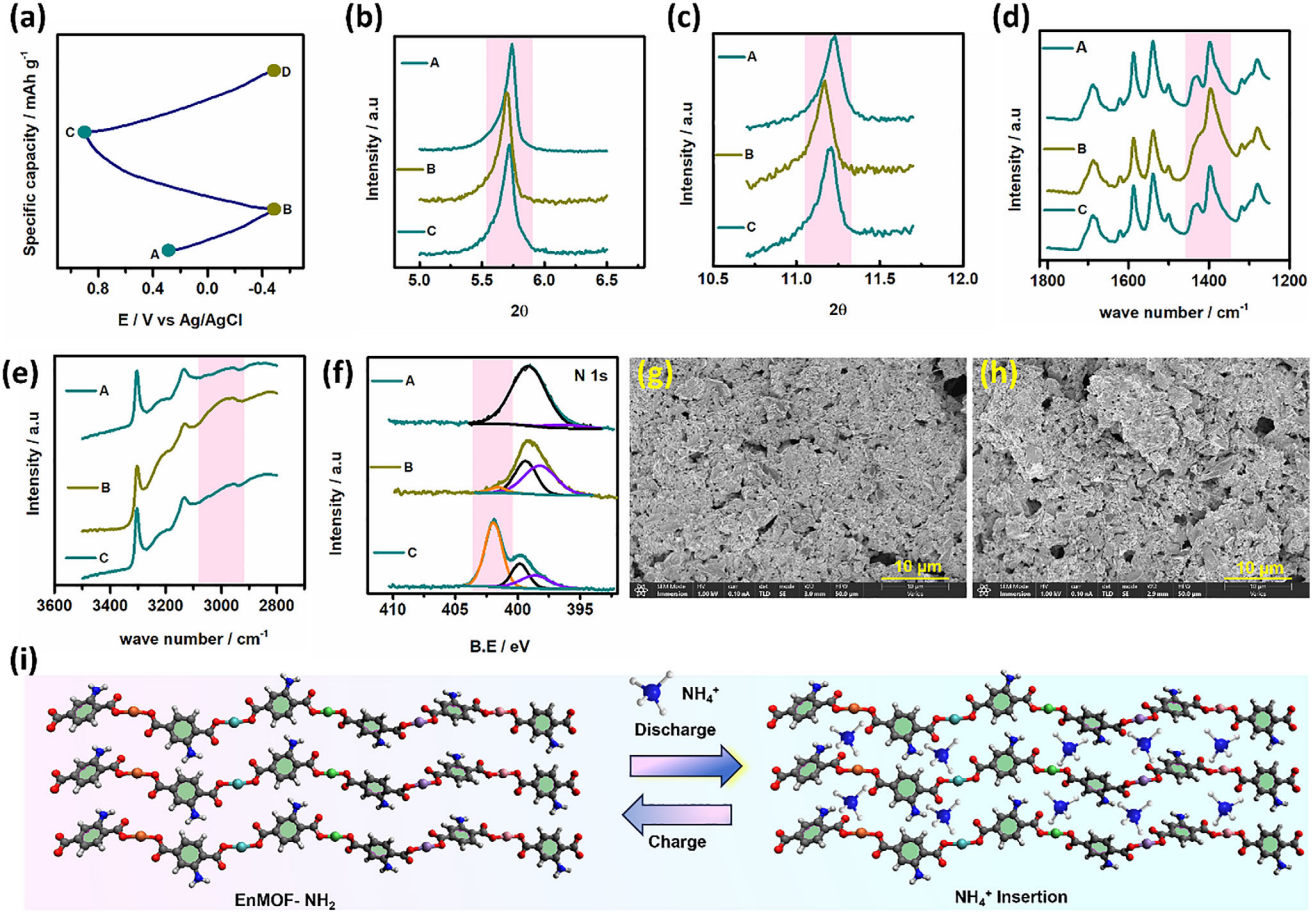
Post characterization of EnMOF‐NH_2_ (a) Charge–discharge profiles at different points (A,B,C,D); (b,c) XRD patterns, (d,e) IR spectra, and (f) XPS spectra of N 1s corresponding to those points; (g,h) SEM images after the first and 1000th cycle, (i) Schematic representation of the NH_4_^+^ ion storage mechanism in EnMOF‐NH_2_ during charge–discharge process.

To understand the structural changes of EnMOF‐NH_2_ during the NH_4_^+^ insertion/extraction process, the ex situ XRD, FTIR, and XPS measurements were carried out at three representative charge‐discharge states (A–C) as shown in Figure 4a. The overall XRD diffraction patterns of EnMOF‐NH_2_ remain stable without showing additional reflections, confirming that EnMOF‐NH_2_ maintains its crystalline framework during cycling (A–C). The two characteristic low‐angle reflections centered at ∼5.72° and ∼11.22° exhibit a systematic shift in 2θ during the NH_4_^+^ insertion/de‐insertion process (Figure 4b,c). Both 2θ values are slightly shifted to lower angles from 5.7246 to 5.6908°, and from 11.2173 to 11.1625° during discharge (state A→B), indicating slight lattice expansion, which might arise from H‐bond‐assisted NH_4_^+^ insertion and local rearrangement in the EnMOF‐NH_2_ framework. The 2θ values shifted back to higher values from 5.6908 to 5.7083°, and 11.1625 to 11.1951° during charge (state B → C), indicating lattice contraction associated with NH_4_^+^ de‐insertion. This reversible lattice expansion and contraction confirms that reversible ammonium ion storage in EnMOF‐NH_2_, which is consistent with previous reports [76, 77].

In addition, *ex situ* FTIR measurements were conducted before and after the charge‐discharge cycles to understand molecular‐level insight into the NH_4_^+^ storage mechanism. The vibrational bands at 1396 cm^−^^1^ and 1434 cm^−^^1^ assigned to carboxylate groups (COO^−^) are broadened and merged during discharge (A→B, Figure 4d), indicating the hydrogen bonding interaction between inserted NH_4_^+^ and framework carboxylate oxygens (NH_4_^+^···O─C═O). Meanwhile, vibrations at 2800 to 3400 cm^−^^1^ region assigned to N–H stretching are significantly broadened and extended to lower wavenumbers (∼3000 cm^−^^1^, Figure 4e), suggesting the hydrogen‐bonding interactions of inserted NH_4_^+^ with the NH_2_ group of framework (NH_4_^+^···NH_2_). These spectral changes are reversed during charging (B→C), in which carboxylate vibrations are separated, and N─H stretching is sharpened. These reversible N─H···O and N─H···N interactions confirm reversible NH_4_^+^ storage within the EnMOF‐NH_2_ during the charge–discharge process.

To further understand the ammonium ion storage mechanism, *ex situ* XPS was recorded at different states (A–C) during the charge‐discharge process. The Co 2p, Ni 2p, Fe 2p, Mn 2p, and Mo 3d spectra of the pristine electrode (A) show spin‐orbit doublets (Figure S14), which are consistent with the expected oxidation states of the transition metal centers in EnMOF‐NH_2_. The spin‐orbit doublets separation at 16.3, 13.5, 11.7, 17.3, and 3.1 eV, assigned to mixed Co(II/III), Fe(II/III), Mn(II/III), Ni(II/III), and Mo(VI) oxidation states, respectively, in line with characteristic 2p/3d splitting reported for these transition metals [78]. There are no significant changes in the spin‐orbit doublet separation during the NH_4_^+^ insertion/de‐insertion process, and no additional doublets, which confirms the preserved oxidation states of the metal nodes and their rigid redox structures.

In contrast, the N 1s spectra show significant changes upon battery cycling (A–C, Figure 4f). The pristine electrode (State A) exhibits a major peak at 399.1 eV related to the NH_2_‐BDC linker of EnMOF‐NH_2_, with minor peak at low B.E [79]. The N 1s spectra were deconvoluted into three contributions at 398.6, 399.4, and 401.6 eV. The new peaks (399.4 and 401.6 eV) might be due to the positively charged nitrogen species arising from inserted NH_4_^+^ during discharge (A→B). The peak profile shifts to a higher binding energy of 401.9 eV, while the 399.8 and 398.6 eV peaks remain, possibly due to oxidation of NH_2_‐BDC linkers at high potentials during charge (B→C). Interestingly, the N 1s profile at the second discharged state (C→D) nearly recovers the first discharged state B (Figure S15), indicating a highly reversible NH_4_^+^ insertion/de‐insertion process with slight irreversible changes in the NH_2_‐BDC linker. The XRD, FTIR, and XPS studies indicate that NH_4_^+^ storage is primarily governed by nitrogen and carboxylate functional groups of EnMOF‐NH_2_ through reversible N─H···N and N─H···O hydrogen bonding. The multimetal nodes in EnMOF‐NH_2_ are structurally intact and exhibit a negligible contribution to NH_4_^+^ storage.

Ex situ SEM was carried out to examine the morphology of the EnMOF‐NH_2_ electrode before and after 1000 charge–discharge cycles (Figure 4g,h). Both electrodes display densely interconnected MOF, without obvious particle fracture or large voids, indicating that the mesoporous framework remains structurally stable after prolonged battery cycling.​ It confirms that highly reversible NH_4_^+^ insertion/extraction occurs within the EnMOF‐NH_2_. This morphological stability and hydrogen‐bonding interactions evidenced by *ex situ* XRD, FTIR, and XPS, underscore the reversible storage of NH_4_^+^ ions within the entropy‐engineered MOF framework. The schematic representation of the NH_4_^+^ storage mechanism in EnMOF‐NH_2_, based on the above studies, is shown in Figure 4i.

### 2.6 Electrochemical Performance of the Full Cell

**FIGURE 5:**
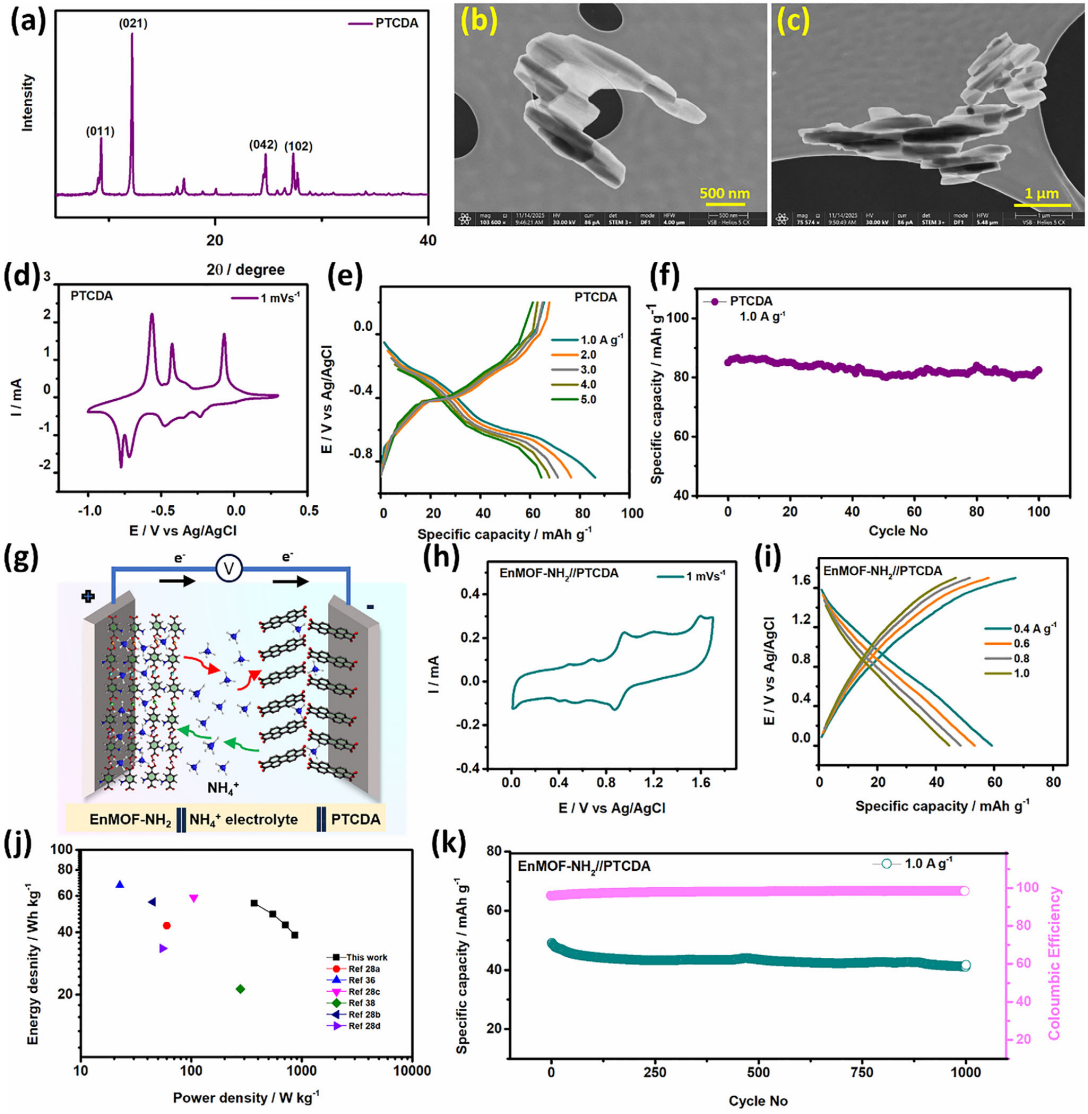
EnMOF‐NH_2_//PTCDA Full cell Performance. Characterization of PTCDA: (a) XRD pattern (b & c) SEM images; Electrochemical performance of the PTCDA half‐cell: (d) cyclic voltammograms, (e) rate capability, and (f) cycling stability. (g) Schematic representation of EnMOF‐NH_2_//PTCDA full cell. (h–k) Electrochemical performance of the EnMOF‐NH_2_//PTCDA full cell: (h) cyclic voltammograms, (i) rate capability, (j) Ragone plot comparing the energy and power densities of our AIB with reported literature values, and (k) cycling stability.

Selecting a suitable anode material for aqueous NH_4_^+^‐ion batteries is essential, which must have a stable electrochemical window before the onset of the hydrogen evolution reaction (HER) [9]. Among various organic anodes, PTCDA is considered a potential organic anode due to its multiple carbonyl redox centers and layered molecular structure [80]. Similar to other perylene‐based organic electrodes, PTCDA can be stabilized by van der Waals interactions within its π‐stacked layers and offers a stable reduction potential, which is suitable for storing NH_4_^+^ ions [81]. Figure 5a–c shows the XRD pattern and SEM images of PTCDA. The XRD pattern of PTCDA displays characteristic diffraction peaks at reflection planes (011), (021), (042), and (102), consistent with a monoclinic β‐phase crystal structure (space group P2_1_/c). This confirms the β‐form of PTCDA prior to electrode fabrication, consistent with previous reports of PTCDA as an anode material in aqueous ammonium‐ion batteries [82, 83]. SEM images show that PTCDA exhibits a rod‐like morphology with smooth surfaces, arising from strong π‐π stacking interactions between the planar perylene cores. This microstructure provides diffusion pathways favorable to the reversible NH_4_^+^ insertion/extraction process, consistent with previous reports [82, 83].

The electrochemical behavior of PTCDA was evaluated by cyclic voltammetry in the potential range of ‐1.1 to +0.3 V vs Ag/AgCl at a scan rate of 1 mV s^−^^1^ (Figure 5d). Cyclic voltammograms of the PTCDA electrode display multiple distinct redox peaks arising from the reversible enolization of its carbonyl groups within the PTCDA framework, indicating its highly reversible NH_4_^+^ insertion/extraction characteristics. This behavior is similar to the reported PTCDI anode in rocking chair AAIBs [68]. The sharp redox feature of PTCDA in the negative potential region is governed by a faradaic‐dominated mechanism, which enables it to pair with EnMOF‐NH_2_ cathode to maximize the full‐cell voltage. The PTCDA electrode is then subjected to GCD studies to examine the NH_4_^+^ storage performance. The corresponding curves display an initial capacity of 86.3 mA h g^−^^1^ at a current density of 1.0 A g^−^^1^, with good rate capability and cycling stability. At current densities of 1.0, 2.0, 3.0, 4.0, and 5.0 A g^−^^1^, the PTCDA electrode delivers discharge capacities of 86.3, 75.6, 71.2, 67.6, and 64.5 mAh g^−^^1^, respectively, and retains about 74.7% of the 1.0 A g^−^^1^ capacity at the highest current density (Figure 5e). The voltage profiles at different current densities show a similar sloping plateau, indicating fast NH_4_^+^ transport with limited polarization. Figure 5f shows the cycling stability of the PTCDA electrode at 1.0 A g^−^^1^. The discharge capacity decreases from 84.9 to 82.4 mAh g^−^^1^ at 1.0 A g^−^^1^ over 100 cycles, retaining 97.0% of its initial capacity. Based on the above half‐cell results, a rocking‐chair–type aqueous NH_4_^+^‐ion full cell was assembled using EnMOF‐NH_2_ as the cathode and PTCDA as the anode with 1.0 M (NH_4_)_2_SO_4_ as the electrolyte. The NH_4_^+^ ions shuttle reversibly between the EnMOF‐NH_2_ cathode and the PTCDA anode during the charge–discharge process, as illustrated in Figure 5g.

The electrochemical behavior of EnMOF‐NH_2_//PTCDA full cell was examined using CV and GCD studies. The full cell was charge‐balanced by adjusting the anode‐to‐cathode mass ratio based on the reversible capacities of the individual electrodes. The CV of the full cell shows multiple distinct redox peaks in the expanded potential window of 0.01 to 1.8 V at 1 mV s^−^^1^ (Figure 5h), which arises from combined redox features of the PTCDA anode and the EnMOF‐NH_2_ cathode. This separation of reduction peaks allows the device to operate over a wide cell voltage while maintaining the electrochemical stability of the aqueous NH_4_^+^ electrolyte. The absence of sharp current spikes at low potentials indicates effective suppression of HER in the two‐electrode configuration, similar to previously reported AAIB full cells [68]. Figure 5i shows the GCD profiles of the EnMOF‐NH_2_//PTCDA full cell at current densities from 0.4 to 1.0 A g^−^^1^. The device delivers a reversible specific capacity of ∼60 mAh g^−^^1^ at a current density of 0.4 A g^−^^1^. Specific capacities of 59.4, 53.7, 48.6, and 44.5 mAh g^−^^1^ are obtained at current densities of 0.4, 0.6, 0.8, and 1.0 A g^−^^1^, respectively, indicating good rate capability. The EnMOF‐NH_2_//PTCDA full cell displays symmetric charge‐discharge curves with reduced voltage hysteresis compared to that of PTCDA anode, indicating its improved electrochemical stability. The EnMOF‐NH_2_//PTCDA full cell achieves a maximum energy density of 55.24 Wh kg^−1^ at a power density of 372 W kg^−1^, as shown in Figure 5j. The achieved energy density and power density are comparable with most of the reported aqueous AIBs including Ni‐APW//PTCDI [68], MnHCF//PTCDI [84], Fe‐MnHCF//PTCDI [85], Mn‐PBA@PPY//PTCDA [70], h‐MoO_3_//CuFe (PBA) [86], NiHCF//AC [69], MnCu‐PBA//BPD [71]. The long‐term cycling of the full cell was demonstrated at 1.0 A g^−^^1^ (Figure 5k). It retains 83% of its initial capacity after 1000 cycles, indicating excellent cycling durability, which is comparable to or better than many reported aqueous NH_4_^+^ full cells based on Prussian‐blue analogues and organic electrodes [10, 68, 72, 87]. The Coulombic efficiency maintains close to 100% throughout the cycling, confirming reversible NH_4_^+^ shuttling between EnMOF‐NH_2_ and PTCDA. Importantly, the performance of this system is not solely related to energy density value, it is a combination of three advantageous factors simultaneously: (i) a wide operating potential window of 0.01 to 1.8 V, enabled by entropy‐engineered EnMOF‐NH_2_ cathode paired with PTCDA; (ii) excellent long‐term cycling stability; (iii) operating in mild 1.0 M (NH_4_)_2_SO_4_ electrolyte. Although several systems in Figure 5j achieve comparable energy densities, they either operate in highly concentrated electrolytes (*e.g*., 5–25 M, Table S4) at significantly low current densities or exhibit inferior cycling stability, which limits their practical applicability. From a practical perspective, the mild aqueous electrolyte, low‐cost organic anode, and scalable synthesis of EnMOF‐NH_2_ collectively position this full cell as a viable candidate for applications where safety and sustainability are prioritized. Further improvements in energy density could be achieved by maximizing active material utilization or by replacing the PTCDA with high‐capacity organic anodes, such as polyimide‐ or anthraquinone‐based materials. Overall, the EnMOF‐NH_2_//PTCDA full cell delivers improved reversible capacity, outstanding cycling stability, and suppressed parasitic reactions. These results demonstrate that pairing organic carbonyl anodes with entropy‐engineered MOF cathodes is a promising strategy for constructing durable and efficient NH_4_^+^‐ion batteries.

## 3 Conclusions

An entropy‐engineered, amino‐functionalized multi‐metallic metal–organic framework (EnMOF‐NH_2_) has been reported as a highly stable and efficient cathode for AAIBs. The integration of five metal nodes with an amino‐functionalized benzene dicarboxylate linker yielded a highly crystalline mesoporous structure with strong metal‐ligand interactions and high porosity, enabling rapid ammonium ion storage via hydrogen bonding. The incorporated Mo metal nodes within the framework serve as stable anchor sites that minimize metal dissolution into the electrolyte during continuous NH_4_^+^ insertion/extraction, enabling outstanding cycling stability and a 162% increase after 2500 cycles. The characterizations (XRD, FTIR, XPS, SEM/TEM, BET, and ICP‐OES) confirm the stable structural integrity, enhanced coordination strength, and minimal metal dissolution of EnMOF‐NH_2_, which is governed by entropy‐engineered multiple redox sites and amino functionalization. Electrochemical kinetics of EnMOF‐NH_2_ reveal fast NH_4_^+^ transport, governed by a mixed diffusion‐surface controlled process, with a dominant capacitive contribution arising from abundant redox active sites and hydrogen bonding interaction. The XRD, IR, and XPS studies reveal that reversible ammonium ion storage occurs via N─H···O and N─H···N hydrogen bonding between NH_4_^+^ ions and amino/carboxylate groups, without structural degradation. Furthermore, a rocking chair full cell comprising EnMOF‐NH_2_ cathode and PTCDA anode enables a wide potential window of 0.01–1.8 V, and displays a specific capacity of 59.4 mAh g^−^^1^ at 0.4 A g^−^^1^, and retains 83% capacity after 1000 cycles with nearly 100% coulombic efficiency. This research highlights entropy‐engineered, amino‐functionalized multi‐metal MOFs as a promising cathode platform for high‐performance AAIBs.

## 4 Experimental Section

### 4.1 Materials

Cobalt (II) nitrate hexahydrate (Co(NO_3_)_2_·6H_2_O, ≥98%), Nickel nitrate hexahydrate (Ni(NO_3_)_2_·6H_2_O, ≥99.99%), Manganese(II) nitrate tetrahydrate (Mn(NO_3_)_2_·4H_2_O, ≥99.99%), Iron(III) nitrate nonahydrate (Fe(NO_3_)_3_·9H_2_O, ≥98%), Ammonium heptamolybdate tetrahydrate ((NH_4_)_6_Mo_7_O_24_·4H_2_O), 2‐amino‐1,4‐benzenedicarboxylic acid (H_2_NC_6_H_3_‐1,4‐(CO_2_H)_2_, 99%), Terephthalic acid (C_6_H_4_‐1,4‐(CO_2_H)_2_, 98%), Ammonium sulfate ((NH_4_)_2_SO_4,_ ≥99.0%), Ammonium acetate (CH_3_CO_2_NH_4_, ≥97%), Ammonium chloride (NH_4_Cl, ≥99.5%), Lithium sulfate (Li_2_SO_4_, 99.5% trace metals basis), Sodium sulfate (Na_2_SO_4_, ≥99.0%), Potassium sulfate (K_2_SO_4_, ≥99.0%), N,N‐Dimethylformamide (DMF, HCON(CH_3_)_2_, ≥99.8%), Activated carbon, Polyvinylidene fluoride ((CH_2_CF_2_)n), N‐Methyl‐2‐pyrrolidone (C_5_H_9_NO, ≥99.0%), Whatman glass microfiber grade (0.67 mm thick, 47 mm diameter), were purchased from Sigma Aldrich. Ethanol was of analytical grade and obtained from P‐LAB, Czech Republic. All materials were of analytical grades unless otherwise stated, used as received without further purification.

### 4.2 Synthesis of EnMOF‐NH_2_

EnMOF‐NH_2_ was synthesized using the solvothermal method using 2‐aminobenzene‐1,4‐dicarboxylic acid (NH_2_‐BDC) as the organic ligand. In particular, 0.25 mmol of each metal salts particularly Co(NO_3_)_2_·6H_2_O, Ni(NO_3_)_2_·6H_2_O, Fe(NO_3_)_3_·9H_2_O, Mn(NO_3_)_2_·4H_2_O, and (NH_4_)_6_Mo_7_O_24_·4H_2_O) were dissolved in a mixture of DMF/ethanol/H_2_O (20:4:4 mL), and the ligand (NH_2_‐BDC) was dissolved into 20 mL DMF. The two solutions were homogeneously mixed under stirring for 45 min, transferred to a Teflon‐lined autoclave, and then subjected to 150°C for 24 h. After cooling to room temperature, the resulting precipitate was centrifuged and washed repeatedly with DMF and ethanol, and dried under vacuum at 60°C. Terephthalic acid (BDC, without NH_2_ functionalization) was used as a ligand under similar conditions to obtain the EnMOF‐BDC.

Control sample without a Mo precursor was synthesized under identical conditions from the same metal salts, except that (NH_4_)_6_Mo_7_O_24_·4H_2_O, using NH_2_‐BDC as the ligand to obtain EnMOF‐NH_2_‐Mo‐free (Co‐Ni‐Fe‐Mn, without Mo). To elucidate the specific contribution of Mo to the electrochemical behavior, additional Mo‐containing systems with lower metal complexity, including binary (CoMo‐NH_2_, NiMo‐NH_2_, FeMo‐NH_2_), ternary (CoFeMo‐NH_2_, CoNiMo‐NH_2_, NiFeMo‐NH_2_), and quaternary (CoNiFeMo‐NH_2_) frameworks, which were also synthesized under the same solvothermal conditions from the corresponding metal precursors and NH_2_‐BDC ligand.

### 4.3 Cathode Preparation

The cathode was fabricated by mixing the active material, conductive carbon, and PVDF binder in a weight ratio of 6:3:1 in N‐methyl‐2‐pyrrolidone (NMP) to obtain a uniform slurry. The slurry was coated onto an exfoliated graphite (EG) current collector and dried under vacuum at 80°C overnight. The total mass loading was approximately 1.0‐1.2 mg cm^−^^2^. The specific capacity values are calculated based on the active material mass. A three‐electrode setup was used for electrochemical characterization, with the prepared cathode as the working electrode, a platinum plate as the counter electrode, and an Ag/AgCl as the reference electrode. An aqueous 1.0 M (NH_4_)_2_SO_4_ solution was used as the electrolyte.

### 4.4 PTCDA Anode

The anode was fabricated by mixing PTCDA, conductive carbon, and PVDF binder in a weight ratio of 7:2:1 in NMP to obtain a homogeneous slurry. The resulting slurry was coated on the EG current collector and dried under vacuum at 120°C for 12 h. An electrochemical test was conducted using a three‐electrode configuration, where PTCDA as the working electrode, a platinum plate as the counter electrode, and Ag/AgCl as the reference electrode, with 1.0 M (NH_4_)_2_SO_4_ aqueous solution as the electrolyte.

### 4.5 Swagelok Two‐Electrode Full Cell

The electrochemical performance was evaluated in a Swagelok two‐electrode configuration, in which EnMOF‐NH_2_ was the cathode, PTCDA was the anode, glass microfiber was the separator, and an aqueous 1.0 M (NH_4_)_2_SO_4_ solution was the electrolyte. This Swagelok cell configuration was employed in cyclic voltammetry and galvanostatic charge–discharge.

### 4.6 Characterization of Materials

XRD patterns of EnMOF‐NH_2_ and control samples were recorded using a Rigaku SmartLab diffractometer (Rigaku, Tokyo, Japan) with Cu Kα radiation (λ = 0.15418 nm). FTIR spectra were recorded using a Thermo Scientific Nicolet iS10 spectrometer equipped with a Smart iTX ATR accessory over the range 4000‐500 cm^−1^ with a spectral resolution of 2 cm^−1^. X‐ray photoelectron spectroscopy (XPS) measurements were carried out using a Thermo Scientific Nexsa G2 system equipped with a monochromated Al Kα X‐ray source (hν = 1486.6 eV). Binding energies for all were referenced to the C 1s peak at 284.8 eV of adventitious carbon present on the surface to account for the charge effect shift. BET analysis was carried out using nitrogen physisorption at 77 K on a 3Flex surface area and porosity analyzer (Micromeritics, USA). Chemical composition of EnMOF‐NH_2_ was determined using ICP‐OES ARCOS MultiView (Spectro, Germany). Morphologies and elemental distributions of the as‐prepared samples were investigated by FE‐SEM equipped with energy‐dispersive X‐ray spectroscopy (EDS, Thermo Fisher Scientific Helios 5 CX DualBeam). TEM images were analyzed by an LVEM 25E—Delong—low voltage transmission electron microscope.

### 4.7 Electrochemical Measurements

The CV studies were conducted on Autolab electrochemical workstation (PGSTAT302N, Metrohm) in the range ‐0.5 to 0.9 V against Ag/AgCl. Galvanostatic discharge‐charge (GCD) tests were performed using a Bi‐Potentiostat SP‐150e (Biologic SAS, France) in a three‐electrode setup over a voltage range of ‐0.5 to 0.9 V vs. Ag/AgCl. Electrochemical impedance spectroscopy (EIS) measurements were conducted in the frequency range of 100 kHz to 10 mHz.

### 4.8 Preparation Protocols for Ex Situ XRD, FTIR, and XPS characterization

The ex situ characterization was conducted in the charge/discharge cycle within the potential window between ‐0.5 to 0.9 V at a current density of 0.5 A g^−1^. Disassemble the three‐electrode setup after discharge/charge to the desired voltages to obtain the corresponding cathode. Then wash the electrode thoroughly with plenty of distilled water. These electrodes were dried at 60°C for 12 h.

## Author Contributions

M.P. and P.B. jointly conceptualized the research idea and designed the projects. P.B. performed material synthesis, characterization, electrochemical testing, data analysis, and drafted the manuscript. M.P. provided direction, supervision, and critical revisions in all stages. The article was developed with contributions from all authors, and all authors have approved the final version.

## Conflicts of Interest

The authors declare no conflicts of interest.

## Supporting information




**Supporting File**: smll74541‐sup‐0001‐SuppMat.docx.

## Data Availability

The data that support the findings of this study are available from the corresponding author upon reasonable request.

## References

[1] F. Zhang , W. Zhang , D. Wexler , and Z. Guo , “Recent Progress and Future Advances on Aqueous Monovalent‐Ion Batteries towards Safe and High‐Power Energy Storage,” Advanced Materials 34 (2022): 2107965, 10.1002/adma.202107965. 35338665

[2] S. Nandi and M. Pumera , “Transition Metal Dichalcogenide‐Based Materials for Rechargeable Aluminum‐Ion Batteries: A Mini‐Review,” Chemsuschem 17 (2024): 202301434, 10.1002/cssc.202301434. 38212248

[3] S. Nandi and M. Pumera , “Materials for Aluminum Batteries: Progress and Challenges,” Chemical Engineering Journal 517 (2025): 164283, 10.1016/j.cej.2025.164283.

[4] S. Nandi , K. K. Sonigara , and M. Pumera , “Exploring the Electrochemistry of Al^3+^ Ion in Amorphous Bi_4_V_2_O_11_ for Rechargeable Aqueous Aluminum‐Ion Battery,” Applied Materials Today 42 (2025): 102568, 10.1016/j.apmt.2024.102568.

[5] P. De , P. Lazar , M. Otyepka , and M. Pumera , “Topological Insulator Bi _2_ Te _3_ Anode for Aqueous Aluminum‐Ion Batteries: Unveiling the Role of Hydronium Ions,” Advanced Science 12 (2025): 07255, 10.1002/advs.202507255. PMC12499396 40619600

[6] K. K. Sonigara , J. V. Vaghasiya , C. C. Mayorga‐Martinez , and M. Pumera , “Flexible Aqueous Zn–S Battery Based on an S‐Decorated Ti_3_C_2_T_x_ Cathode,” npj 2D Materials and Applications 7 (2023): 45, 10.1038/s41699-023-00411-2.

[7] K. K. Sonigara , J. V. Vaghasiya , C. C. Mayorga‐Martinez , and M. Pumera , “Point‐of‐Use Upcycling of 3D Printing Waste for Developing 3D‐printed Zn–I _2_ Batteries,” Journal of Materials Chemistry A 13 (2025): 11804–11816, 10.1039/D5TA00919G.

[8] K. K. Sonigara , J. V. Vaghasiya , and M. Pumera , “Corrosion‐Resistant Shape‐Programmable Zn–I _2_ Battery,” Advanced Energy Materials 15 (2025): 2401321, 10.1002/aenm.202401321.

[9] Y. Wang and S. F. Kuchena , “Recent Progress in Aqueous Ammonium‐Ion Batteries,” ACS Omega 7 (2022): 33732–33748, 10.1021/acsomega.2c04118. 36188297 PMC9520733

[10] J. Han , A. Varzi , and S. Passerini , “The Emergence of Aqueous Ammonium‐Ion Batteries,” Angewandte Chemie International Edition 61 (2022): 202115046, 10.1002/anie.202115046. PMC9303650 34913235

[11] S. Nandi and M. Pumera , “Topological Insulator Layered Bi _2_ Te _3_ Based 3D‐Printed Nanocarbon Electrode for Rechargeable Aqueous Ammonium‐Ion Battery,” Advanced Functional Materials 35 (2025): 2506723, 10.1002/adfm.202506723.

[12] J. Liu , X. Kuang , M. Ge , and A. Sun , “Research Progress of Cathode Materials for Ammonium‐Ion Batteries,” Journal of Energy Storage 110 (2025): 115209, 10.1016/j.est.2024.115209.

[13] Y. Pan , L. Yuan , L. Liu , et al., “Critical Advances of Aqueous Rechargeable Ammonium Ion Batteries,” Small Structures 4 (2023): 2300201, 10.1002/sstr.202300201.

[14] X. Zhang , M. Xia , H. Yu , et al., “Hydrogen Bond‐Assisted Ultra‐Stable and Fast Aqueous NH^4+^ Storage,” Nano‐Micro Letters 13 (2021): 139, 10.1007/s40820-021-00671-x. 34138392 PMC8192672

[15] P. Zhou , J. Lv , X. Huang , Y. Lu , and G. Wang , “Strategies for Enhancing the Catalytic Activity and Electronic Conductivity of MOFs‐Based Electrocatalysts,” Coordination Chemistry Reviews 478 (2023): 214969, 10.1016/j.ccr.2022.214969.

[16] T. Sun , Z. Wang , L. Zeng , and G. Feng , “Identifying MOFs for Electrochemical Energy Storage via Density Functional Theory and Machine Learning,” npj Computational Materials 11 (2025): 90, 10.1038/s41524-025-01590-w.

[17] A. K. K. Padinjareveetil , J. V. Perales‐Rondon , D. Zaoralová , M. Otyepka , O. Alduhaish , and M. Pumera , “Fe‐MOF Catalytic Nanoarchitectonic Toward Electrochemical Ammonia Production,” ACS Applied Materials & Interfaces 15 (2023): 47294–47306, 10.1021/acsami.3c12822. 37782845 PMC10571008

[18] A. Terzopoulou , M. Palacios‐Corella , C. Franco , et al., “Biotemplating of Metal–Organic Framework Nanocrystals for Applications in Small‐Scale Robotics,” Advanced Functional Materials 32 (2022): 2107421, 10.1002/adfm.202107421.

[19] Y. Ying , B. Khezri , J. Kosina , and M. Pumera , “Reconstructed Bismuth‐Based Metal−Organic Framework Nanofibers for Selective CO _2_ ‐to‐Formate Conversion: Morphology Engineering,” Chemsuschem 14 (2021): 3402–3412, 10.1002/cssc.202101122. 34227725

[20] Y. Ying , M. P. Browne , and M. Pumera , “Metal–Organic‐Frameworks on 3D‐Printed Electrodes: In Situ Electrochemical Transformation Towards the Oxygen Evolution Reaction,” Sustainable Energy & Fuels 4 (2020): 3732–3738, 10.1039/D0SE00503G.

[21] Y. Ying , A. M. Pourrahimi , Z. Sofer , S. Matějková , and M. Pumera , “Radioactive Uranium Preconcentration via Self‐Propelled Autonomous Microrobots Based on Metal–Organic Frameworks,” ACS Nano 13 (2019): 11477–11487, 10.1021/acsnano.9b04960. 31592633

[22] B. Khezri and M. Pumera , “Metal–Organic Frameworks Based Nano/Micro/Millimeter‐Sized Self‐Propelled Autonomous Machines,” Advanced Materials 31 (2019): 1806530, 10.1002/adma.201806530. 30701595

[23] W. Lu , D. De Alwis Jayasinghe , M. Schröder , and S. Yang , “Ammonia Storage in Metal–Organic Framework Materials: Recent Developments in Design and Characterization,” Accounts of Materials Research 5 (2024): 1279–1290, 10.1021/accountsmr.4c00183. 39478984 PMC11519835

[24] X. Zeng , J. Li , M. He , et al., “High Adsorption of Ammonia in a Titanium‐based Metal–Organic Framework,” Chemical Communications 60 (2024): 5912–5915, 10.1039/D4CC01449A. 38712387

[25] C. Li , H. Zhang , M. Liu , F.‐F. Lang , J. Pang , and X.‐H. Bu , “Recent Progress in Metal–Organic Frameworks (MOFs) for Electrocatalysis,” Industrial Chemistry & Materials 1 (2023): 9–38, 10.1039/D2IM00063F.

[26] W. Zheng and L. Y. S. Lee , “Metal–Organic Frameworks for Electrocatalysis: Catalyst or Precatalyst?,” ACS Energy Letters 6 (2021): 2838–2843, 10.1021/acsenergylett.1c01350.

[27] Basree , A. Ali , K. Kumari , M. Ahmad , and G. C. Nayak , “Functional Metal–Organic Frameworks Derived Electrode Materials for Electrochemical Energy Storage: A Review,” Chemical Communications 60 (2024): 13292–13313, 10.1039/D4CC04086D. 39465622

[28] H. B. Wu and X. W. Lou , “Metal‐organic Frameworks and Their Derived Materials for Electrochemical Energy Storage and Conversion: Promises and Challenges,” Science Advances 3 (2017): aap9252, 10.1126/sciadv.aap9252. PMC5714063 29214220

[29] M. Ahmad , J. Chen , J. Liu , et al., “Metal‐Organic Framework‐Based Single‐Atom Electro‐/Photocatalysts: Synthesis, Energy Applications, and Opportunities,” Carbon Energy 6 (2023): 382, 10.1002/cey2.382.

[30] Z. A. Sandhu , M. A. Raza , N. S. Awwad , et al., “Metal–organic Frameworks for Next‐Generation Energy Storage Devices; a Systematic Review,” Materials Advances 5 (2024): 30–50, 10.1039/D3MA00822C.

[31] Z. Fan , J. Wang , Y. Wu , and P. Zhang , “Advanced High‐Entropy Materials for High‐Quality Energy Storage and Conversion,” Energy Storage Materials 74 (2025): 103954, 10.1016/j.ensm.2024.103954.

[32] X. Mu , M. Yu , X. Liu , et al., “High‐Entropy Ultrathin Amorphous Metal–Organic Framework‐Stabilized Ru(Mo) Dual‐Atom Sites for Water Oxidation,” ACS Energy Letters 9 (2024): 5763–5770, 10.1021/acsenergylett.4c02552.

[33] X. Xiao , S. Tao , H. Lian , et al., “Unveiling the Microscopic Origin of Ultrahigh Initial Coulombic Efficiency of High‐Entropy Metal–Organic Frameworks for Sodium Storage,” ACS Nano 18 (2024): 28444–28455, 10.1021/acsnano.4c11401. 39356953

[34] S. Xu , M. Li , H. Wang , et al., “High‐Entropy Metal–Organic Framework Arrays Boost Oxygen Evolution Electrocatalysis,” The Journal of Physical Chemistry C 126 (2022): 14094–14102, 10.1021/acs.jpcc.2c05083.

[35] L. Liu , Z. Cai , S. Yang , et al., “Multifunctional High‐Entropy Alloy Nanolayer toward Long‐Life Anode‐Free Sodium Metal Battery,” Advanced Materials 37 (2025): 2413331, 10.1002/adma.202413331. 39610214

[36] Y. Ma , Y. Ma , S. L. Dreyer , et al., “High‐Entropy Metal–Organic Frameworks for Highly Reversible Sodium Storage,” Advanced Materials 33 (2021): 2101342, 10.1002/adma.202101342. 34245051 PMC11469271

[37] J. Su , Y. Wan , L. Feng , et al., “One‐Stone, Two‐Birds″: Zinc‐Rich Metal–Organic Frameworks as Precursors for High‐entropy Zn‐air Battery Electrocatalysts with Hierarchical Pore Structures,” Angewandte Chemie International Edition 64 (2025): 202413826, 10.1002/anie.202413826. 39198219

[38] J. S. R. Fernando , S. S. Asaithambi , and S. M. Chavan , “Amino‐functionalizing Ce‐based MOF UiO‐66 for Enhanced CO_2_ Adsorption and Selectivity,” ChemPlusChem 89 (2024): 202400107, 10.1002/cplu.202400107. 38708570

[39] Y. Wan , Y. Miao , T. Qiu , et al., “Tailoring Amine‐functionalized Ti‐MOFs via a Mixed Ligands Strategy for High‐efficiency CO_2_ Capture,” Nanomaterials 11 (2021): 3348, 10.3390/nano11123348. 34947697 PMC8709442

[40] D. Bahamon , W. Anlu , S. Builes , M. Khaleel , and L. F. Vega , “Effect of Amine Functionalization of MOF Adsorbents for Enhanced CO_2_ Capture and Separation: A Molecular Simulation Study,” Frontiers in Chemistry 8 (2020): 574622, 10.3389/fchem.2020.574622. 33585395 PMC7873881

[41] L. Yaqoob , T. Noor , N. Iqbal , H. Nasir , and A. Mumtaz , “Electrocatalytic Performance of NiNH_2_BDC MOF Based Composites with rGO for Methanol Oxidation Reaction,” Scientific Reports 11 (2021): 13402, 10.1038/s41598-021-92660-8. 34183691 PMC8238968

[42] A. Justin , J. Espín , I. Kochetygov , M. Asgari , O. Trukhina , and W. L. Queen , “A Two Step Postsynthetic Modification Strategy: Appending Short Chain Polyamines to Zn‐NH _2_ ‐BDC MOF for Enhanced CO _2_ Adsorption,” Inorganic Chemistry 60 (2021): 11720–11729, 10.1021/acs.inorgchem.1c01216. 34264652

[43] M. Ma , E. Chen , H. Yue , G. Tian , and S. Feng , “Entropy Engineering Activation of UiO‐66 for Boosting Catalytic Transfer Hydrogenation,” Nature Communications 16 (2025): 367, 10.1038/s41467-024-52225-5. PMC11699162 39753533

[44] H. B. Wu , B. Y. Xia , L. Yu , X.‐Y. Yu , and X. W. Lou , “Porous Molybdenum Carbide Nano‐octahedrons Synthesized via Confined Carburization in Metal‐Organic Frameworks for Efficient Hydrogen Production,” Nature Communications 6 (2015): 6512, 10.1038/ncomms7512. PMC4382699 25758159

[45] W. Lu , Z. Wei , Z.‐Y. Gu , et al., “Tuning the Structure and Function of Metal–Organic Frameworks via Linker Design,” Chemical Society Review 43 (2014): 5561–5593, 10.1039/C4CS00003J. 24604071

[46] B. A. Berehe , A. A. Desalew , G. W. Derbe , et al., “Enhanced Photocatalytic Degradation of Methylene Blue Dye via Valorization of a Polyethylene Terephthalate Plastic Waste‐Derived Metal–Organic Framework‐based ZnO@Co‐BDC Composite Catalyst,” Nanoscale Advances 7 (2025): 3834–3845, 10.1039/D4NA01071J. 40401279 PMC12091243

[47] X. Hu , H. Wang , S. Qi , et al., “Co/C Nanomaterial Derived from Co Metal–Organic Framework for Oxygen Evolution Reaction,” Ionics 28 (2022): 813–821, 10.1007/s11581-021-04376-4.

[48] J. Zhai , X. Ma , J. Zhao , Y. Xiong , and Y. Tao , “Nickel‐based Metal‐Organic‐Frameworks as Electrocatalysts for Electrochemical Oxidation of Ammonia,” Applied and Computational Engineering 70 (2024): 92–101, 10.54254/2755-2721/70/20240962.

[49] Z. Liu , W. He , Q. Zhang , H. Shapour , and M. F. Bakhtari , “Preparation of a GO/MIL‐101(Fe) Composite for the Removal of Methyl Orange from Aqueous Solution,” ACS Omega 6 (2021): 4597–4608, 10.1021/acsomega.0c05091. 33644567 PMC7905816

[50] L. T. Tran , H. T. M. Dang , H. V. Tran , G. T. L. Hoang , and C. D. Huynh , “MIL‐88B(Fe)‐NH 2: An Amine‐Functionalized Metal–Organic Framework for Application in a Sensitive Electrochemical Sensor for Cd ^2+^ , Pb ^2+^ , and Cu ^2+^ Ion Detection,” RSC Advances 13 (2023): 21861–21872, 10.1039/D3RA02828C. 37475762 PMC10354696

[51] Z. Ye , R. Oriol , C. Yang , I. Sirés , and X.‐Y. Li , “A Novel NH_2_‐MIL‐88B(Fe)‐Modified Ceramic Membrane for the Integration of Electro‐Fenton and Filtration Processes: A Case Study on Naproxen Degradation,” Chemical Engineering Journal 433 (2022): 133547, 10.1016/j.cej.2021.133547.

[52] S. Sundriyal , V. Shrivastav , M. Sharma , S. Mishra , and A. Deep , “Redox Additive Electrolyte Study of Mn–MOF Electrode for Supercapacitor Applications,” ChemistrySelect 4 (2019): 2585–2592, 10.1002/slct.201900305.

[53] C. E. Castañeda‐Calzoncit , D. A. Castañeda‐Medina , J. A. Claudio‐Rizo , C. M. Sánchez‐Cruz , and D. A. López‐Badillo , “Biocompatible Molybdenum Complexes Based on Terephthalic Acid and Derived from PET: Synthesis and Characterization,” Asian Journal of Applied Science and Technology 6 (2022): 25–34, 10.38177/ajast.2022.6304.

[54] K. L. Timofeev , S. A. Kulinich , and T. S. Kharlamova , “NH_2_‐Modified UiO‐66: Structural Characteristics and Functional Properties,” Molecules 28 (2023): 3916, 10.3390/molecules28093916. 37175325 PMC10180438

[55] A. Ali‐Ahmad , T. Hamieh , T. Roques‐Carmes , M. Hmadeh , and J. Toufaily , “Effect of Amino Functional Groups on the Surface Properties and Lewis's Acid Base Parameters of UiO‐66(NH2) by Inverse Gas Chromatography,” Heliyon 10 (2024): e23839, 10.1016/j.heliyon.2023.e23839. 38226281 PMC10788446

[56] Y. Wu , P. Hu , F. Xiao , et al., “Metal‐Organic Frameworks Based Single‐atom Catalysts for Advanced Fuel Cells and Rechargeable Batteries,” Journal of Energy Chemistry 80 (2023): 501–534, 10.1016/j.jechem.2023.01.054.

[57] D. Bara , E. G. Meekel , I. Pakamorė , C. Wilson , S. Ling , and R. S. Forgan , “Exploring and Expanding the Fe‐Terephthalate Metal–Organic Framework Phase Space by Coordination and Oxidation Modulation,” Materials Horizons 8 (2021): 3377–3386, 10.1039/D1MH01663F. 34665190 PMC8628537

[58] M. J. Remy , A. C. Vieira Coelho , and G. Poncelet , “Surface Area and Microporosity of 1.8 Nm Pillared Clays from the Nitrogen Adsorption Isotherm,” Microporous Materials 7 (1996): 287–297, 10.1016/S0927-6513(96)00021-1.

[59] A. Galarneau , F. Villemot , J. Rodriguez , F. Fajula , and B. Coasne , “Validity of the T‐plot Method to Assess Microporosity in Hierarchical Micro/Mesoporous Materials,” Langmuir 30 (2014): 13266–13274, 10.1021/la5026679. 25232908

[60] P. Bazylewski , D. W. Boukhvalov , A. I. Kukharenko , et al., “The Characterization of Co‐Nanoparticles Supported on Graphene,” RSC Advances 5 (2015): 75600–75606, 10.1039/C5RA12893E.

[61] J. Xing , Y. Liu , G. Mathew , et al., “High‐Entropy Metal–Organic Frameworks and Their Derivatives: Advances in Design, Synthesis, and Applications for Catalysis and Energy Storage,” Advanced Science 12 (2025): 2411175, 10.1002/advs.202411175. 39665155 PMC11792049

[62] G. Wang , Z. Chen , J. Zhu , et al., “High‐Entropy Amorphous Catalysts for Water Electrolysis: A New Frontier,” Nano‐Micro Letters 18 (2025): 77, 10.1007/s40820-025-01936-5. 41111133 PMC12535958

[63] C. Wang , G.‐T. Sun , C.‐Y. He , B.‐H. Liu , Z.‐W. Lu , and X.‐H. Gao , “Entropy‐Driven Multiphase Engineering Enables Superior Broadband Infrared Emissivity in High‐Entropy Oxides,” Advanced Materials 38 (2025): 08636, 10.1002/adma.202508636. 40955707

[64] D. M. D'Alessandro , “Exploiting Redox Activity in Metal–Organic Frameworks: Concepts, Trends and Perspectives,” Chemical Communications 52 (2016): 8957–8971, 10.1039/C6CC00805D. 26988560

[65] R. Shah , S. Ali , S. Ali , et al., “Amino Functionalized Metal‐Organic Framework/rGO Composite Electrode for Flexible Li‐Ion Batteries,” Journal of Alloys and Compounds 936 (2023): 168183, 10.1016/j.jallcom.2022.168183.

[66] Z. Tian , V. S. Kale , Y. Wang , et al., “High‐Capacity NH ^4+^ Charge Storage in Covalent Organic Frameworks,” Journal of the American Chemical Society 143 (2021): 19178–19186, 10.1021/jacs.1c09290. 34739750

[67] Y. Yang , R. Lin , L. Ge , et al., “Synthesis and Characterization of Three Amino‐Functionalized Metal–Organic Frameworks Based on the 2‐aminoterephthalic Ligand,” Dalton Transactions 44 (2015): 8190–8197, 10.1039/C4DT03927K. 25848648

[68] X. Wu , Y. Qi , J. J. Hong , Z. Li , A. S. Hernandez , and X. Ji , “Rocking‐Chair Ammonium‐Ion Battery: A Highly Reversible Aqueous Energy Storage System,” Angewandte Chemie International Edition 56 (2017): 13026–13030, 10.1002/anie.201707473. 28859240

[69] M. Zhou , T. Wu , M. Kang , et al., “Ligand Field‐Induced Dual Active Sites Enhance Redox Potential of Nickel Hexacyanoferrate for Ammonium Ion Storage,” Advanced Materials 37 (2025): 2419446, 10.1002/adma.202419446. 40391638 PMC12355446

[70] Q. Liu , D. Zhang , Y. Yang , et al., “Encapsulation of Prussian Blue Analogues with Conductive Polymers for High‐Performance Ammonium‐Ion Storage,” Advanced Energy Materials 15 (2025): 2402863, 10.1002/aenm.202402863.

[71] Y. Shi , H. Qiu , Z. Xu , et al., “Boosting Performance of Aqueous Ammonium Ion Batteries via Mn‐Partial‐Substituted Cu Prussian Blue Cathode,” ACS Sustainable Chemistry & Engineering 13 (2025): 14162–14169, 10.1021/acssuschemeng.5c06135.

[72] C.‐Y. Wei , Z.‐H. Sun , Z.‐Y. Gu , D.‐X. Han , L. Niu , and X.‐L. Wu , “Ultralong‐Life Aqueous Ammonium‐Ion Batteries Enabled by Unlocking Inert‐Site of Medium‐Entropy Prussian Blue Analogs,” Advanced Energy Materials 15 (2025): 2500589, 10.1002/aenm.202500589.

[73] J. Yang , W. Wang , Z. Cao , et al., “Self‐Polymerization of Carbonyl Pigments for High‐Performance Aqueous Ammonium‐ion Batteries,” Chemical Communications 61 (2025): 5499–5502, 10.1039/D5CC00011D. 40099554

[74] Y.‐Z. Zhang , J. Liang , Z. Huang , et al., “Ionically Conductive Tunnels in h ‐WO _3_ Enable High‐Rate NH _4_ ^+^ Storage,” Advanced Science 9 (2022): 2105158, 10.1002/advs.202105158. 35107225 PMC8981906

[75] F. Long , L. Zhang , and Y. Gao , “Enabling a High‐Entropy Effect Paradigm for Efficient Zn ^2+^ /NH _4_ ^+^ Energy Storage,” Advanced Energy Materials 15 (2025): 04021, 10.1002/aenm.202504021.

[76] C. Han , J. Zhu , K. Fu , D. Deng , W. Luo , and L. Mai , “A High‐Capacity Polyaniline‐Intercalated Layered Vanadium Oxide for Aqueous Ammonium‐Ion Batteries,” Chemical Communications 58 (2022): 791–794, 10.1039/D1CC05677H. 34927651

[77] F. Ye , R. Pang , C. Lu , et al., “Reversible Ammonium Ion Intercalation/De‐Intercalation with Crystal Water Promotion Effect in Layered VOPO_4_·2H_2_O,” Angewandte Chemie International Edition 62 (2023): 202303480, 10.1002/anie.202303480. 37041737

[78] Q. Lu , “How to Correctly Analyze 2p X‐Ray Photoelectron Spectra of 3d Transition‐Metal Oxides: Pitfalls and Principles,” ACS Nano 18 (2024): 13973–13982, 10.1021/acsnano.4c03964. 38776459

[79] L. Shi , T. Wang , H. Zhang , et al., “An Amine‐Functionalized Iron(III) Metal–Organic Framework as Efficient Visible‐Light Photocatalyst for Cr(VI) Reduction,” Advanced Science 2 (2015): 1500006, 10.1002/advs.201500006. 27980927 PMC5115284

[80] M. Karlsmo , R. Bouchal , and P. Johansson , “High‐Performant All‐Organic Aqueous Sodium‐Ion Batteries Enabled by PTCDA Electrodes and a Hybrid Na/Mg Electrolyte,” Angewandte Chemie International Edition 60 (2021): 24709–24715, 10.1002/anie.202111620. 34528364 PMC8596776

[81] Q. Zhao , Z. Zhu , and J. Chen , “Molecular Engineering with Organic Carbonyl Electrode Materials for Advanced Stationary and Redox Flow Rechargeable Batteries,” Advanced Materials 29 (2017): 1607007, 10.1002/adma.201607007. 28370809

[82] S. Krishnan , D. K. Rai , and X. Wang , “Boosting the Performance of Aqueous Ammonium‐ion Batteries by Mitigating Side Reactions Using Polymer Additive,” ACS Applied Polymer Materials 5 (2023): 9274–9285, 10.1021/acsapm.3c01739.

[83] T. Ogawa , K. Kuwamoto , S. Isoda , T. Kobayashi , and N. Karl , “3,4:9,10‐Perylenetetracarboxylic Dianhydride (PTCDA) by Electron Crystallography,” Acta Crystallographica Section B Structural Science 55 (1999): 123–130, 10.1107/S0108768198009872. 10927346

[84] H. Zhang , Y. Tian , W. Wang , Z. Jian , and W. Chen , “Organic Ammonium Ion Battery: A New Strategy for a Nonmetallic Ion Energy Storage System,” Angewandte Chemie International Edition 61 (2022): 202204351, 10.1002/anie.202204351. 35470508

[85] L. Du , S. Bi , M. Yang , Z. Tie , M. Zhang , and Z. Niu , “Coupling Dual Metal Active Sites and Low‐solvation Architecture toward High‐performance Aqueous Ammonium‐ion Batteries,” Proceedings of the National Academy of Sciences 119 (2022): 2214545119, 10.1073/pnas.2214545119. PMC9897483 36472961

[86] G. Liang , Y. Wang , Z. Huang , et al., “Initiating Hexagonal MoO _3_ for Superb‐Stable and Fast NH _4_ ^+^ Storage Based on Hydrogen Bond Chemistry,” Advanced Materials 32 (2020): 1907802, 10.1002/adma.201907802. 32080917

[87] T. Wang , X. Li , S. Zhao , et al., “Rocking‐Chair Ammonium Ion Battery with High Rate and Long‐Cycle Life,” Chinese Chemical Letters 35 (2024): 108449, 10.1016/j.cclet.2023.108449.

