# Supplementary material for: Entropy‐Engineered Multi‐Metallic MOFs Unlock High‐Performance Ammonium‐Ion Storage
Source: Small. 2026 Jul 27;22(45):e74541. doi: 10.1002/smll.74541 (PMC13472302; doi:10.1002/smll.74541)
Supplement: Supplementary file 1 [file SMLL-22-e74541-s001.docx]

**Entropy-Engineered Multi-Metallic MOFs Unlock High-Performance Ammonium-Ion Storage**

Palani Barathi^a^, Ľubica Cenknerová^b^, and Martin Pumera^a,c,d,e^*

^a^Advanced Nanorobots and Multiscale Robotics Laboratory, Faculty of Electrical Engineering and Computer Science, VSB - Technical University of Ostrava, 17. listopadu 2172/15, Ostrava 708 00, Czech Republic

^b^Analytical laboratory, Institute of Organic Chemistry and Biochemistry of the Czech Academy of Sciences, Flemingovo náměstí 542/2, 160 00 Praha 6, Czech Republic

^c^Future Energy and Innovation Laboratory, Central European Institute of Technology, Brno University of Technology, Purkynova 123, Brno 61200, Czech Republic

^d^Department of Medical Research, China Medical University Hospital, China Medical University, No. 91 Hsueh-Shih Road, Taichung, Taiwan, China

^e^Department of Chemical and Biomolecular Engineering, Yonsei University, 50 Yonsei-ro, Seodaemun-gu, Seoul 03722, Korea

* author for correspondence, M. Pumera; E-mail: [martin.pumera@vsb.cz](mailto:martin.pumera@vsb.cz)

**Figure S1.** HRSEM (a,b) and TEM (c) images of EnMOF-BDC

**(a)**

**(b)**

**Figure S2.** STEM–EDS elemental mapping of (a) EnMOF-NH_2_ and (b) EnMOF-BDC.

**Table S1.** Elemental composition (STEM–EDS) of EnMOF-NH_2_ and EnMOF-BDC.

| Element | EnMOF-NH₂ (point, %) | EnMOF-BDC (point, %) |
| --- | --- | --- |
| Co | 17.8 | 17.7 |
| Ni | 6.7 | 20.3 |
| Fe | 28.9 | 15.2 |
| Mn | 20.0 | 17.7 |
| Mo | 26.7 | 29.1 |

**Figure S3.** N₂ adsorption–desorption analysis of EnMOF-NH₂ and EnMOF-BDC. (a,b) N₂ adsorption–desorption isotherms (77 K); (c,d) t-plots for surface-area evaluation.

**Table S2.** BET textural parameters of EnMOF-NH₂ and EnMOF-BDC.

| **Sample** | **BET surface area (m² g⁻¹)** | **External surface areaᵃ (m² g⁻¹)** | **Micropore volume (cm³ g⁻¹)** | **Total pore volumeᵇ (cm³ g⁻¹)** | **Average pore diameterᶜ (nm)** |
| --- | --- | --- | --- | --- | --- |
| **EnMOF-NH₂** | 23.1 | 23.3 | 0.000056 | 0.086 | 3–10 |
| **EnMOF-BDC** | 14.0 | 15.8 | –0.000688 | 0.046 | 3–8 |

ᵃ From t-plot analysis. ᵇ From BJH desorption data. ᶜ Main mesopore range.

**Figure S4.** XPS survey spectrum of EnMOF and EnMOF-NH_2_

**Table S3.** Elemental composition (XPS) of EnMOF-NH_2_ and EnMOF-BDC.

| Element | EnMOF-NH₂ (point, %) | EnMOF-BDC (point, %) |
| --- | --- | --- |
| Co | 16.52 | 17.59 |
| Ni | 6.74 | 12.76 |
| Fe | 34.22 | 32.79 |
| Mn | 12.97 | 13.91 |
| Mo | 29.57 | 22.94 |

**Figure S5.** Rate capability (a), capacity retention (b), and (c) cycling stability of EnMOF-BDC.

**Table S4.** Summary of various cathode materials and the corresponding performance for AIBs.

| **Materials** | **Electrolyte** | **Potential Range** | **Capacity (mAh/g)** | **Cycle life (Capacity retention)** | **Ref** |
| --- | --- | --- | --- | --- | --- |
| Ni-APW | 1.0 M (NH_4_)_2_SO_4_ | 0.2–0.9 V *vs.* Ag/AgCl | 60 at 0.15 A g^-1^ | 74% after 2000 cycles at 0.3 A g^-1^ | ^1^ |
| MnHCF | 1.0 M NH_4_TFSI in TEGDME | 0–1.0 V *vs.* Ag/AgCl | 104 at 0.1 A g^-1^ | 98% after 100 cycles at 0.1 A g^-1^ | ^2^ |
| FeMnHCF | 24.0 M NH_4_Otf | -0.5–1.3 V *vs*. Ag/AgCl | 123.8 at 0.5 A g^-1^ | 68.5% after 10,000cycles at 3 A g^-1^ | ^3^ |
| Mn-PBA@ppy | 2.0 M NH_4_Otf | -0.2–1.0 V *vs*. Ag/AgCl | 72 at 0.1 A g^-1^ | 94% after 300 cycles at 0.1 A g^-1^ | ^4^ |
| N-HEPBA | 1 M (NH_4_)_2_SO_4_ + 5 mM CuSO_4_ | 0–1.2 V *vs*. Ag/AgCl | 129 at 0.1 A g^-1^ | 100% after 1000 cycles at 2 A g^-1^ | ^5^ |
| MnO_x_ | 0.5 M NH_4_Ac | 0–0.8 V *vs*. SCE | 176 at 0.5 A g^-1^ | 94.7% after 10,000 cycles at 5 A g^-1^ | ^6^ |
| Mg_0.5_Zn_0.5_Mn_2_O_4_ | 1.0 M (NH_4_)_2_SO_4_ | 0–1.1 V *vs*. SCE | 113.7 at 2.0 A g^-1^ | 60% after 600 cycles at 2 A g^-1^ | ^7^ |
| VS_2_/Vo_x_ | 5.0 M (NH_4_)_2_SO_4_ | -0.6–0.9 V *vs*. Ag/AgCl | 150 at 0.1 A g^-1^ | 43% after 1000 cycles at 1 A g^-1^ | ^8^ |
| (NH_4_)_2_V_10_O_25_·8H_2_O | 1 M NH_4_Cl/PVA | -0.8–1.0 V *vs*. Ag/AgCl | 171 at 0.5 A g^-1^ | 100% after 10,000 cycles at 1 A g^-1^ | ^9^ |
| PVO | 0.5 M (NH_4_)_2_SO_4_ | -0.5–0.9 V *vs*. SCE | 307 at 0.5 A g^-1^ | 42% after 100 cycles at 5 A g^-1^ | ^10^ |
| MoO_3_ | 1.0 M NH_4_Cl | -0.5–0.8 V *vs*. SCE | 115 at 0.1 A g^-1^ | 94% after 100,000 cycles at 15 A g^-1^ | ^11^ |
| MoO_x_ | 1.0 M NH_4_Ac | -1.0–0 V *vs*. SCE | 175 at 0.22 A g^-1^ | 72.6% after 3000 cycles at 2.2 A g^-1^ | ^12^ |
| h-WO_3_ | 1.0 M (NH_4_)_2_SO_4_ | -1.1–0.8 V *vs.* Ag/AgCl | 82 at 1 A g^-1^ | 68% after 200,000 cycles at 20 A g^-1^ | ^13^ |
| PNNI | 1.0 M NH_4_Ac | -0.9–0.2 V *vs*. SCE | 147.7 at 0.1 A g^-1^ | 90.7% after 2000 cycles at 5A g^-1^ | ^14^ |
| Polypyrrole | 25.0 M NH_4_Ac | -1.0–0.5 V *vs.* Ag/AgCl | 125 at 1 A g^−1^ | 73.77% after 100 cycles at 10 A g^-1^ | ^15^ |
| DAAQ-TP-COF | 1.0 M NH_4_Ac | -0.7–0.1 V *vs.* Ag/AgCl | 141 at 0.1 A g^-1^ | 90% after 8000 cycles at 6 A g^-1^ | ^16^ |
| P420 | 1 M (NH_4_)_2_SO_4_ | -0.8–0.2 V *vs.* Ag/AgCl | 98.8 at 0.1 A g^-1^ | 86% after 200 cycles at 1 A g^-1^ | ^17^ |
| CNCMF-PBAs | 5.8 M (NH_4_)_2_SO_4_ | 0–1.05 *vs.* Ag/AgCl | 80 at 0.2 A g^-1^ | 223.5% after 45000 cycles at 2 A g^-1^ | ^18^ |
| ZnCuHCF | 23 M NH_4_OTf + 0.5 M Zn(OTf)_2_ | 0–1.05 *vs.* Ag/AgCl | 121.7 at 1 A g^-1^ | 92.1% after 10 000 cycles at 6 A g^–1^ | ^19^ |
| NiHCF | 2 M NH_4_Ac | 0–0.8 V vs SCE | 61.3 at 0.05 A g^-1^ | 71.1% after 1000 cycles at 1 A g^–1^ | ^20^ |
| MnCu-PBA | 1 M NH_4_Cl | 0.3–1.1 *vs.* Ag/AgCl | 78 at 0.1 Ag^-1^ | 100% after 10000 cycles at 2 A g^–1^ | ^21^ |
| EnMOF-NH_2_ | 1M (NH_4_)_2_SO_4_ | -0.5–0.9 V vs Ag/AgCl | 130.8 at 0.3 Ag^-1^ | 162% after 2500 cycles at 1 Ag^-1^ | This work |

Ni-APW, (NH_4_)_1.47_Ni[Fe(CN)_6_]_0.88;_ MnHCF, manganese hexacyanoferrate; NH_4_TFSI, Bis(trifluoromethane)sulfonimide ammonium; TEGDM, Tetraethylene glycol dimethyl ether; FeMnHCF, Fe-substituted Mn-based PBA; NH_4_Otf, Ammonium Triflate, Mn-PBA@ppy, coated Mn-PBA with conductive polymers polypyrrole; N-HEPBA, high-entropy PBA; MnO*_x_*, manganese oxides; MoO_x_, molybdenum oxides; PVO, polyaniline (PANI) intercalated V_2_O_5_; PVA, polyvinyl alcohol; PNNI, poly(1,4,5,8-naphthalenetetracarboxylic anhydride naphthylamine) imine; DAAQ-TP-COF, anthraquinone carbonyl-containing covalent-organic framework; P420, perylene-3,4,9,10-tetracarboxylic dianhydride; CNCMF-PBAs, Cu, Ni, Co, Mn, and Fe at the 4b sites in PBAs; ZnCuHCF, Zn-doping dual-active-site CuHCF; NiHCF, Nickel hexacyanoferrate; MnCu-PBA, Mn-partially substituted Cu-based Prussian blue analogues.

**Table S5.** ICP-OES results of electrolyte after 100 cycles showing metal ion dissolution for EnMOF-NH_2_ and CoNiFeMn-NH₂ electrodes.

| **Sample** | **Element mg/kg** | | | | |
| --- | --- | --- | --- | --- | --- |
|  | **Co** | **Ni** | **Fe** | **Mn** | **Mo** |
|  |  |  |  |  |  |
| EnMOF-NH_2_ | 6.23 | 2.24 | 4.84 | 5.60 | 8.21 |
| CoNiFeMn-NH₂ | 29.51 | 34.30 | 12.80 | 22.22 | - |

**Figure S6.** Cyclic voltammograms, rate capability, capacity retention at different current densities of CoMo-NH_2_ (a,b,c); NiMo-NH_2_ (d,e,f) and FeMo-NH_2_ (g,h,i) in 1 M (NH_4_)_2_SO_4_ aqueous electrolyte.

**Figure S7.** Cyclic voltammograms, rate capability, capacity retention at different current densities of CoFeMo-NH_2_ (a,b,c); CoNiMo-NH_2_ (d,e,f) and NiFeMo-NH_2_ (g,h,i) in 1 M (NH_4_)_2_SO_4_ aqueous electrolyte.

**Figure S8.** (a) Cyclic voltammograms, (b) rate capability, (c) capacity retention at different current densities of CoNiFeMo-NH_2_ in 1 M (NH_4_)_2_SO_4_ aqueous electrolyte

**Figure S9.** Cyclic voltammograms, rate capability, capacity retention at different current densities of EnMOF-NH_2_ in (a,b,c)1M Li_2_SO_4_ ; (d,e,f) 1M Na_2_SO_4_ and (g,h,i) 1M K_2_SO_4_ aqueous electrolytes.

**Figure S10.** Comparison of capacities with respect to cycle no. at a current density of 0.3 A g^-1^ in 1 M (NH_4_)_2_SO_4_; 1M Li_2_SO_4_ ;1M Na_2_SO_4_ and 1M K_2_SO_4_ aqueous electrolytes.

**Figure S11.** Cyclic voltammograms of EnMOF-NH_2_ at a scan rate of 1 mVs^-1^ and comparison of capacities with respect to cycle no. at a current rate of 0.3 A g^-1^ in 1 M (NH_4_)_2_SO_4_, 1 M NH_4_Ac, and 1 M NH_4_Cl aqueous electrolytes.

**Figure S12.** Cyclic voltammograms and GCD curves of EnMOF-NH_2_ at a scan rate of 20 mVs^-1^ and at a current rate of 0.5 A g^-1^, in 1 M (NH_4_)_2_SO_4_ under different pH conditions.

**Figure S13.** (a) Cyclic voltammograms of EnMOF-NH_2_ at different scan rates in 1 M (NH_4_)_2_SO_4_ aqueous electrolyte, (b) double logarithmic plot of peak current versus scan rate, (c) diffusive and capacitive contribution ratios of EnMOF-NH_2_ at different scan rates.

**Figure S14.** *Ex situ* XPS analysis of the EnMOF-NH_2_ electrode before and after electrochemical cycling. Comparison of the Co 2p, Ni 2p, Fe 2p, Mn 2p, and Mo 3d core-level spectra for the electrode in the pristine state (A), after NH₄⁺ insertion (B), and after NH₄⁺ de-insertion (C). The dashed lines mark the characteristic binding energies associated with different oxidation states of the metal centers.

**Figure S15.** *Ex situ* XPS analysis of N 1s at the point state D.

**Details of Calculations:**

(1) **Configuration Entropy Calculation**

For a solid solution, the configurational entropy per mole can be calculated based on the following equation:^[2]^

$\Delta S_{conf}=-R\sum_{i=1}^{n} x_{i}{lnx}_{i}$	(Equation S1)

Where R is the ideal gas constant and $x_{i}$ is 0.2, represents the molar fraction of the i component.

$\Delta S_{conf}=-R\left( 0.2\times\ln\left( 0.2 \right) \right)\times5=1.609 R$

| **System** | **n** | **xᵢ (mole fraction)** | **ΔS_conf_** |
| --- | --- | --- | --- |
| **Binary** | 2 | 0.5 each | R ln 2 = 0.693R |
| **Ternary** | 3 | 0.333 each | R ln 3 = 1.099R |
| **Quaternary** | 4 | 0.25 each | R ln 4 = 1.386R |
| **Quinary** | 5 | 0.2 each | R ln 5 = 1.609R |

(2) **b-value**

The current response of the cyclic voltammetry curve at different voltage value and sweep speed follow the following equation:

$i={av}^{b}$	(Equation S2)

Where *i* is the current response value (mA), *v* is the sweep speed (mV s^-1^), and *a* and *b* are suitable values.

(3) **Capacitance contribution**

The capacity contribution can be quantified according to the following equation:

$i=k_{1}v+k_{1}v^{1/2}$	(Equation S3)

Where *k_1_* value was obtained from the slope of linear fit of the *i/v^1/2^* versus *v^1/2^* plot. The capacitive and diffusion-controlled contributions were obtained by *k_1_v* and *k_2_v^1/2^*, respectively.

**Supporting Information:** Morphology and structural characterization of EnMOF-BDC (Figure S1); STEM-EDS elemental mapping (Figure S2, Table S1); N₂ adsorption–desorption analysis (Figure S3, Table S2); XPS survey spectra and elemental composition (Figure S4, Table S3); Electrochemical performance of EnMOF-BDC (Figure S5); Comparison table of cathode materials (Table S4); ICP-OES dissolution data (Table S5); Rate capability of binary, ternary, quaternary MOFs (Figures S6–S8); Electrolyte comparison studies (Figures S9–S11); pH-dependent electrochemical study (Figure S12); Kinetics analysis (Figure S13); Ex-situ XPS data (Figures S14–S15).

**References**

1. X. Wu, Y. Qi, J. J. Hong, Z. Li, A. S. Hernandez, X. Ji, “Rocking-chair ammonium-ion battery: a highly reversible aqueous energy storage system,” *Angewandte Chemie International Edition* 56 (2017): 13026–13030. https://doi.org/10.1002/anie.201707473
2. H. Zhang, Y. Tian, W. Wang, Z. Jian, W. Chen, “Organic ammonium ion battery: a new strategy for a nonmetallic ion energy storage system,” *Angewandte Chemie International Edition* 61 (2022): e202204351. https://doi.org/10.1002/anie.202204351
3. L. Du, S. Bi, M. Yang, Z. Tie, M. Zhang, Z. Niu, “Coupling dual metal active sites and low-solvation architecture toward high-performance aqueous ammonium-ion batteries,” *Proceedings of the National Academy of Sciences of the United States of America* 119 (2022): e2214545119. https://doi.org/10.1073/pnas.2214545119
4. Q. Liu, D. Zhang, Y. Yang, Y. Gu, Z. Liang, W. Chen, Y. Wu, L. Hu, “Encapsulation of Prussian blue analogues with conductive polymers for high-performance ammonium-ion storage,” *Advanced Energy Materials* 15 (2025): 2402863. https://doi.org/10.1002/aenm.202402863
5. Y. Shen, J. Zou, H. Lan, Y. Ding, Z. Liang, Z. Yang, Z. Zeng, J. Long, Y. Zhao, L. Fu, et al., “Unlocking Prussian blue analogues inert-site to achieve high-capacity ammonium storage,” *Advanced Functional Materials* 34 (2024): 2400598. https://doi.org/10.1002/adfm.202400598
6. Y. Song, Q. Pan, H. Lv, D. Yang, Z. Qin, M.-Y. Zhang, X. Sun, X.-X. Liu, “Ammonium-ion storage using electrodeposited manganese oxides,” *Angewandte Chemie International Edition* 60 (2021): 5718–5722. https://doi.org/10.1002/anie.202013110
7. S. Li, L. Tong, B. Zhang, X. Fu, “Hollow MnO₂ nanospheres containing Mg and Zn for aqueous NH₄⁺ storage: implications for ammonium-ion batteries,” *ACS Applied Nano Materials* 7 (2024): 13745–13755. https://doi.org/10.1021/acsanm.4c02394
8. D. Yu, Z. Wei, X. Zhang, Y. Zeng, C. Wang, G. Chen, Z. X. Shen, F. Du, “Boosting Zn²⁺ and NH₄⁺ storage in aqueous media via in situ electrochemical induced VS₂/VOₓ heterostructures,” *Advanced Functional Materials* 31 (2021): 2008743. https://doi.org/10.1002/adfm.202008743
9. X. Tan, F. Zhang, D. Chen, P. Wang, Y. Liu, C. Meng, Y. Zhang, “Modulating NH₄⁺ in vanadium oxide framework for high-efficient aqueous NH₄⁺ storage,” *Chemical Engineering Journal* 489 (2024): 151119. https://doi.org/10.1016/j.cej.2024.151119
10. C. Han, J. Zhu, K. Fu, D. Deng, W. Luo, L. Mai, “A high-capacity polyaniline-intercalated layered vanadium oxide for aqueous ammonium-ion batteries,” *Chemical Communications* 58 (2022): 791–794. https://doi.org/10.1039/D1CC05677H
11. G. Liang, Y. Wang, Z. Huang, F. Mo, X. Li, Q. Yang, D. Wang, H. Li, S. Chen, C. Zhi, “Initiating hexagonal MoO₃ for superb-stable and fast NH₄⁺ storage based on hydrogen bond chemistry,” *Advanced Materials* 32 (2020): 1907802. https://doi.org/10.1002/adma.201907802
12. Y. Lin, L. Ta, J. Meng, Y. Song, X.-X. Liu, “Electrodepositing amorphous molybdenum oxides for aqueous NH₄⁺ storage,” *Chemical Communications* 59 (2023): 1481–1484. https://doi.org/10.1039/D2CC06450B
13. Y.-Z. Zhang, J. Liang, Z. Huang, Q. Wang, G. Zhu, S. Dong, H. Liang, X. Dong, “Ionically conductive tunnels in h-WO₃ enable high-rate NH₄⁺ storage,” *Advanced Science* 9 (2022): 2105158. https://doi.org/10.1002/advs.202105158
14. S. Zhang, K. Zhu, Y. Gao, D. Cao, “A long cycle stability and high rate performance organic anode for rechargeable aqueous ammonium-ion battery,” *ACS Energy Letters* 8 (2023): 889–897. https://doi.org/10.1021/acsenergylett.2c01962
15. S. F. Kuchena, Y. Wang, “A metal-free all-organic ammonium-ion battery with low-temperature applications,” *Journal of Materials Chemistry A* 11 (2023): 2814–2825. https://doi.org/10.1039/D2TA08988B
16. J. Liu, K. Guo, W. Guo, J. Chang, Y. Li, F. Bao, “Superconjugated anthraquinone carbonyl-based covalent organic framework as anode material for high-performance aqueous ammonium-ion batteries,” *Angewandte Chemie International Edition* 64 (2025): e202424494. https://doi.org/10.1002/anie.202424494
17. J. Yang, W. Wang, Z. Cao, J. Zhang, H. Ren, Y. Yang, H. Shao, S. Dong, “Self-polymerization of carbonyl pigments for high-performance aqueous ammonium-ion batteries,” *Chemical Communications* 61 (2025): 5499–5502. https://doi.org/10.1039/D5CC00011D
18. C.-Y. Wei, Z.-H. Sun, Z.-Y. Gu, D.-X. Han, L. Niu, X.-L. Wu, “Ultralong-life aqueous ammonium-ion batteries enabled by unlocking inert-site of medium-entropy Prussian blue analogs,” *Advanced Energy Materials* 15 (2025): 2500589. https://doi.org/10.1002/aenm.202500589
19. L. Han, Y. Ling, W. Gong, D. Xiao, J. Luo, Q. Zhang, “In situ dynamic compensation strategy enables Jahn–Teller effect suppression in Zn-doped dual-active-site copper hexacyanoferrate cathodes for high-energy and ultra-stable ammonium-ion storage,” *Angewandte Chemie International Edition* 64 (2025): e202507427. https://doi.org/10.1002/anie.202507427
20. M. Zhou, T. Wu, M. Kang, T. Cheng, H. Li, L. He, C. Lian, T. Ma, Q. Zhao, “Ligand field-induced dual active sites enhance redox potential of nickel hexacyanoferrate for ammonium ion storage,” *Advanced Materials* 37 (2025): 2419446. https://doi.org/10.1002/adma.202419446
21. Y. Shi, H. Qiu, Z. Xu, M. Chen, H. Gao, X. Yu, J. Xu, J. Cao, “Boosting performance of aqueous ammonium ion batteries via Mn-partial-substituted Cu Prussian blue cathode,” *ACS Sustainable Chemistry & Engineering* 13 (2025): 14162–14169. https://doi.org/10.1021/acssuschemeng.5c06135
